# Assessing the cognitive status of *Drosophila* by the value-based feeding decision

**DOI:** 10.1038/s41514-021-00075-6

**Published:** 2021-09-15

**Authors:** Chih-Chieh Yu, Ferng-Chang Chang, Yong-Huei Hong, Jian-Chiuan Li, Po-Lin Chen, Chun-Hong Chen, Tzai-Wen Chiu, Tsai-Te Lu, Yun-Ming Wang, Chih-Fei Kao

**Affiliations:** 1 Institute of Molecular Medicine and Bioengineering, National Yang Ming Chiao Tung University, Hsinchu, Taiwan; 2 Center for Intelligent Drug Systems and Smart Bio-devices (IDS2B), National Yang Ming Chiao Tung University, Hsinchu, Taiwan; 3 Department of Biological Science and Technology, College of Biological Science and Technology, National Yang Ming Chiao Tung University, Hsinchu, Taiwan; 4 Institute of Biomedical Engineering, National Tsing Hua University, Hsinchu, Taiwan; 5 National Institute of Infectious Diseases and Vaccinology, National Health Research Institutes, Miaoli County, Taiwan

**Keywords:** Neuroscience, Ageing

## Abstract

Decision-making is considered an important aspect of cognitive function. Impaired decision-making is a consequence of cognitive decline caused by various physiological conditions, such as aging and neurodegenerative diseases. Here we exploited the value-based feeding decision (VBFD) assay, which is a simple sensory–motor task, to determine the cognitive status of *Drosophila*. Our results indicated the deterioration of VBFD is notably correlated with aging and neurodegenerative disorders. Restriction of the mushroom body (MB) neuronal activity partly blunted the proper VBFD. Furthermore, using the *Drosophila* polyQ disease model, we demonstrated the impaired VBFD is ameliorated by the dinitrosyl iron complex (DNIC-1), a novel and steady nitric oxide (NO)-releasing compound. Therefore we propose that the VBFD assay provides a robust assessment of *Drosophila* cognition and can be used to characterize additional neuroprotective interventions.

## Introduction

Decision-making is the act of choosing between available options in facing a need or a problem. The process of decision-making usually involves several steps, including the identification of a need and its potential options, evaluation of options, decision-making and acting, and finally a review of the decision that may assist the prospective decision-making when a similar need/problem is encountered. Decision-making is generally considered as a high-level cognitive process^1,2^. The most intriguing part of the decision-making process is the evaluation of accessible options, which may be based on values, preferences, and past experience of the decision maker. The underlying process and neural circuits of decision-making are currently topics of intense study. A common experimental paradigm for elucidating neural decision-making in mammals is the two-alternative forced choice task (2AFC). Two alternative options are concurrently presented to the test subject. Within a defined time, the test subject has to choose between two alternatives. Distinct features of the two alternatives and creative experimental designs have been exploited to study the specific behavior dynamics of choice under different physiological conditions and, most importantly, the involved neural elements.

Similar two-choice assays have been adapted by the *Drosophila* model to study diverse aspects of the feeding behavior (i.e., two-choice feeding assay)^3–5^. Such studies have allowed scientists to achieve substantial progress in understanding the neuronal and molecular mechanisms that modulate feeding decisions. Particularly, feeding decisions are implemented in the nervous system on multiple levels, from the peripheral chemosensory organs to the central brain^3,6–8^. Many neuronal and molecular mechanisms regulating insect feeding decisions have been uncovered^9–12^. Interestingly, previous studies have demonstrated wild-type (WT) fruit flies are able to differentiate the nutritional values of two sugar solutions and learn to associate an odorant paired with the nutritious sugar solution^3,6,7^. The advantageous decision for most young WT flies to ingest the metabolizable sugar, but not the non-metabolizable sugar, allows the starved flies to quickly regain the nutritional homeostasis. Most importantly, the efficacy of feeding decision based on the caloric contents of two sugar solutions is robust and no pre-conditioning is required. To be able to make the proper feeding decision underscores the neural mechanisms governing the decision-making process, which is regarded as a unique process of cognitive function. The efficacy of proper feeding decision may therefore be used to promptly and faithfully evaluate the cognitive status of fruit flies.

In this study, we explored the efficacy of value-based feeding decision (VBFD) in *Drosophila* under selected physiological conditions. Our results indicated the efficacy of VBFD is susceptible to the chronological ages, alterations of life expectancy, and neurodegenerative disorders. The characterization of simple and complex VBFDs also enabled us to investigate distinct levels of *Drosophila* cognitive function and identify neural circuits related to the making of VBFD. Lastly, we further demonstrated the VBFD assay is an effective approach to identify compounds that have protective functions to disease-associated cognitive disorders.

## Results

### Deterioration of proper VBFD in the aged *Drosophila*

**Fig. 1:**
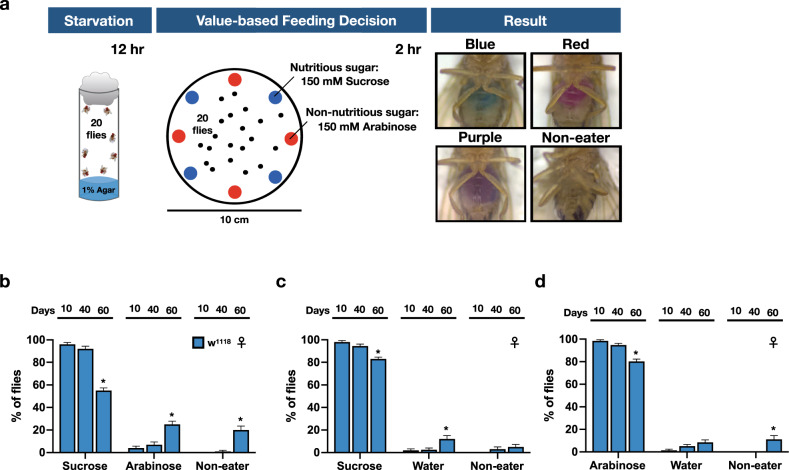
The efficacy of making proper VBFD is gradually deteriorating in aging flies. **a** A schematic illustration of VBFD assay. Briefly, food-deprived flies (12 h of starvation) were presented with two liquid food choices and allowed to feed for 2 h at 25 °C. Each liquid food is labeled by a red or a blue dye. The feeding decision was scored by examining the colors shown in the abdomen. Red or Blue color shown in the abdomen suggests flies only ingest only either one food choice. Purple indicates the flies consume both food choices during the feeding assay. Non-eater means the flies have no labeled food in the digestive tract. **b**–**d** VBFD assays were performed in female flies of different chronological ages: 10, 40, and 60 days. The food choices used were 150 mM sucrose and 150 mM arabinose in (**b**); 150 mM sucrose and plain water in (**c**); 150 mM arabinose and plain water in (**d**). Results were expressed as means ± SEM and analyzed by two-way ANOVA. *n* = 100 for each condition. *p < 0.01. The statistical significance was assessed by the comparison to 10-day-old flies. All flies used in above assays were maintained at 25 °C.

To meet the acute nutritional needs, fast and reliable mechanisms are necessary for fruit flies to evaluate the nutritional values of food substances and determine the subsequent feeding behavior. That is to keep on eating the same food source or switch to another food source with higher nutritional value. Making such VBFD relies on the proper function of central brain mechanisms, which may represent a unique spectrum of cognitive processes. To demonstrate VBFD, the two-choice feeding assay was used to determine the feeding decision of fruit flies^3–5^. Briefly, in this assay, food-deprived fruit flies were presented with two sugar solutions, the metabolizable sucrose and the non-metabolizable arabinose. The relative nutritional values of two sugars were verified by their life-supporting capability as the sole source of food (Supplementary Fig. 1a). Each sugar solution was colored with a blue or a red dye, and the feeding decision was scored by the dye accumulation in the abdomen of individual flies (Fig. 1a). This quantitative measurement of feeding decision indicated the starved 10-day-old w^1118^ adult flies exhibit a strong preference for the metabolizable sucrose (92–97.5% of total tested flies) over the non-metabolizable arabinose (2.5–8%), suggesting young w^1118^ flies can efficiently evaluate the caloric contents of sugars and make appropriate VBFD within the short feeding period (Fig. 1b). Moreover, the capability to make proper VBFD was equally effective in both genders (Fig. 1b and Supplementary Fig. 1c). Next, we asked if VBFD remains intact in the aged flies. The survival analysis revealed the median lifespan of w^1118^ flies is ~63 days in females and ~68 days in males (Supplementary Fig. 1b). We therefore chose two additional time points before the median lifespan to characterize the efficacy of VBFD. Intriguingly, while 40-day-old starved flies recognized and preferred the nutritious sucrose, this capability was significantly deteriorating in 60-day-old flies of both sexes (Fig. 1b and Supplementary Fig. 1c). The population of flies consuming only the non-nutritious arabinose significantly increased to 7–10.5% in 40-day-old flies and to 23–25% in 60-day-old flies. Furthermore, we also noted, as aging progresses, the population of Non-eater flies also increased (from 0% in 10-day-old flies to 11–20% in 60-day-old flies), suggesting this phenotype may be associated with aging. However, when directly measuring the food intake of aged flies, we found most aged flies were highly motivated by the presence of food substance and consume more sucrose solution in response to 12 h of starvation (Supplementary Fig. 2). Therefore, the Non-eater flies appeared insensitive to the hunger-driven signals. Next, to further explore if the decline of making appropriate VBFD is caused by the inability to sense sugar, we assayed the feeding decision between the sweet sugar solution (sucrose or arabinose) and plain water. Unlike the VBFD between the sucrose and arabinose, the sweet sensation and the preference to either sweet sugar were much less affected by aging, as evident by the high percentage of sugar-ingesting population observed in the aged flies (Fig. 1c, d and Supplementary Fig. 1d, e). Presumably, the feeding decision to consume the sweet sugar over water appears to be simpler than the decision relied on the capability to differentiate nutritional values of sugar solutions. We like to consider these two feeding decisions as simple and complex VBFD, respectively. In addition to the w^1118^ flies, similar VBFD results were obtained using a WT fly strain, the Canton-S flies (Supplementary Fig. 3). Like the young w^1118^ flies, the 10-day-old Canton-S flies exhibited high efficiency to make the proper complex and simple VBFDs (in both genders, more than 98% of tested flies ingested the sucrose solution; Supplementary Fig. 3b–d). Moreover, the marked decline of the complex VBFD was observed in the aged Canton-S flies (60-day-old; the age was very close to the median lifespan of Canton-S flies), as evident by the reduction of fly population (from >98% to <75%) consuming only the nutritious sucrose solution and the increase of arabinose-ingesting and Non-eater flies (Supplementary Fig. 3b). In terms of the sweet sensation, more than ~80% of aged Canton-S flies still maintained the ability to recognize the signals of sweetness and make the proper simple VBFD (Supplementary Fig. 3c, d), suggesting the sweet sensation of Canton-S flies is less affected by the aging process. Intriguingly, in comparison to the age-matched w^1118^ flies, the efficacy of making proper complex VBFD was slightly higher in the 60-day-old aged Canton-S flies (compare Fig. 1b and Supplementary Fig. 3b). Therefore, it is likely that the *white* gene may have some effects on the neural circuits governing feeding decisions^13^. However, more studies were needed for further validation. Taken together, our results indicated the ability to differentiate nutritional values of sugar solutions is conserved among distinct fly strains and the age-dependent deterioration of VBFD may not be directly linked to the loss of sweet sensation. Instead, the impaired VBFD may be associated with the decline of cognitive function.

### VBFD in flies that have altered life expectancy

**Fig. 2:**
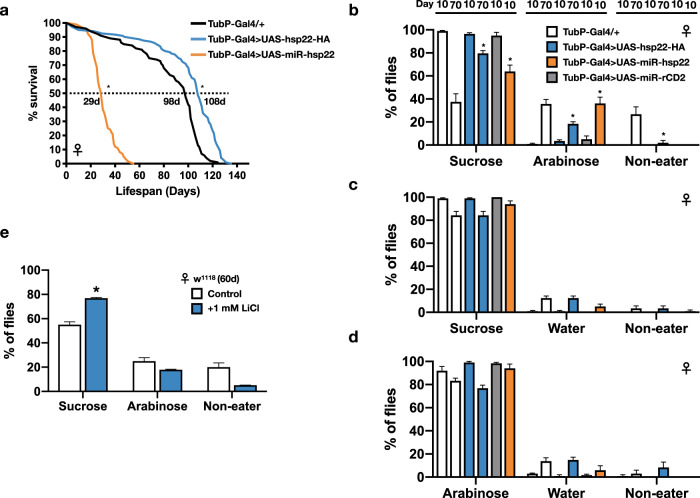
Aged long-lived flies still maintain the proper VBFD. **a** Survival curves of Hsp22 over-expressing (OE; UAS-hsp22-HA) and knockdown (KD; UAS-miR-hsp22) flies. Results were analyzed by the log-rank test. *n* = 100 for each genotype. *p < 0.01. **b**–**d** VBFD assays were performed in female flies with altered life expectancy mediated by up- or down-regulating the expression of *Drosophila* Hsp22. Flies of two chronological ages were used: 10 and 70 days. The food choices used were 150 mM sucrose and 150 mM arabinose in (**b**); 150 mM sucrose and plain water in (**c**); and 150 mM arabinose and plain water in (**d**). Results were expressed as means ± SEM and analyzed by two-way ANOVA. *n* = 100 for each condition. *p < 0.01. The statistical significance was assessed by the comparison to age-matched controls. **e** Female w^1118^ flies fed with 1 mM LiCl for 60 days were given the choices between 150 mM sucrose and 150 mM arabinose in the VBFD assay. Results were expressed as means ± SEM and analyzed by two-way ANOVA. The statistical significance was assessed by the comparison to age-matched controls. *p < 0.01. *n* = 100 for w^1118^ w/o LiCl and *n* = 40 for w^1118^ w/ LiCl. Note that the column of w^1118^ w/o LiCl treatment was the same as presented in Fig. 1b. Genotypes: control (TubP-Gal4/+ and UAS-miR-rCD2/+; TubP-GAL4/+); OE (UAS-hsp22-HA/+; TubP-Gal4/+); KD (UAS-miR-hsp22/+; TubP-Gal4/+). All flies used in above assays were maintained at 25 °C.

Given the decision-making is regarded as a basic cognitive process^1,2^, we used the VBFD assay to assess the cognitive status of *Drosophila* subjected to selected physiological/pathological conditions. For example, there are various genetic manipulations that effectively alter the life expectancy of *Drosophila*. It is especially interesting to know if the aged long-lived flies could still preserve the functional cognition. Studies by Morrow et al.^14^ have shown ectopic expression of Hsp22 (heat-shock protein 22) significantly increases both the median and maximal lifespan of fruit flies. Here, the Hsp22-mediated long-lived flies were assayed to determine their efficacy of VBFD making. Our survival analysis indicated the median lifespan of Hsp22-expressing female flies was ~108 days (Fig. 2a). Therefore, VBFD assays were performed in 10-day- and 70-day-old female flies. As the population of sucrose-ingesting flies declined drastically in the 70-day-old control flies, ~80% of the age-matched long-lived flies still made the proper VBFD by ingesting only sucrose, but not the arabinose (Fig. 2b). However, the efficacy of proper complex VBFD was eventually declined in the 90-day-old long-lived female flies (Supplementary Fig. 4a). Less than 60% of 90-day-old female long-lived flies chose the sucrose, albeit this percentage was still higher than 70-day-old control flies. Therefore, our results suggest the pro-longevity effects elicited by ectopic Hsp22 partly slow down the aging-related cognitive decline. In comparison to the VBFD between nutritious and non-nutritious sugars, feeding decisions between the sweet sugar and plain water, which could be considered as a simpler form of VBFD, were less affected in both the age-matched control and long-lived female flies (Fig. 2c, d; results of male flies are shown in Supplementary Fig. 4d–g). Nonetheless, the fact that 90-day-old long-lived female flies were unable to differentiate the sweet sugars from the plain water suggests the sweet sensation may not be fully functional even in the presence of pro-longevity effects (Supplementary Fig. 4b, c). Together, our results indicate the Hsp22-elicited pro-longevity effects are able to improve the maintenance of cognition in aged flies. Surprisingly, we found expression of Hsp22 specifically in neurons was sufficient to extend the lifespan of fruit flies (Supplementary Fig. 5a, b) and delay the age-dependent impairment of VBFD (Supplementary Fig. 5c–e). These results also further highlight the healthy nervous system is closely related to performance of cognition.

Contrary to the pro-longevity effects, down-regulating Hsp22 expression through the developmental stages drastically reduced the life expectancy of flies (Fig. 2a; UAS-miR-hsp22). Intriguingly, while the complex VBFD was jeopardized in the 10-day-old Hsp22-knockdown female flies, the recognition of sweet sucrose and arabinose remained intact, suggesting this anti-longevity effects have a significant impact on the complex VBFD but not the basic sweet sensation (Fig. 2b–d). Expression of the control microRNAs (UAS-miR-rCD2), by contrast, had little or no effects on the efficacy of VBFD. In addition to the genetic manipulations, lithium chloride (LiCl)-treated long-lived flies (Supplementary Fig. 6) were also able to maintain the proper VBFD when they are aged^15^ (Fig. 2e). In summary, the two pro-longevity treatments we tested not only extend the life expectancy but also have the capability to preserve the cognitive function in aged flies.

### VBFD in flies that have neurodegenerative disorders

**Fig. 3:**
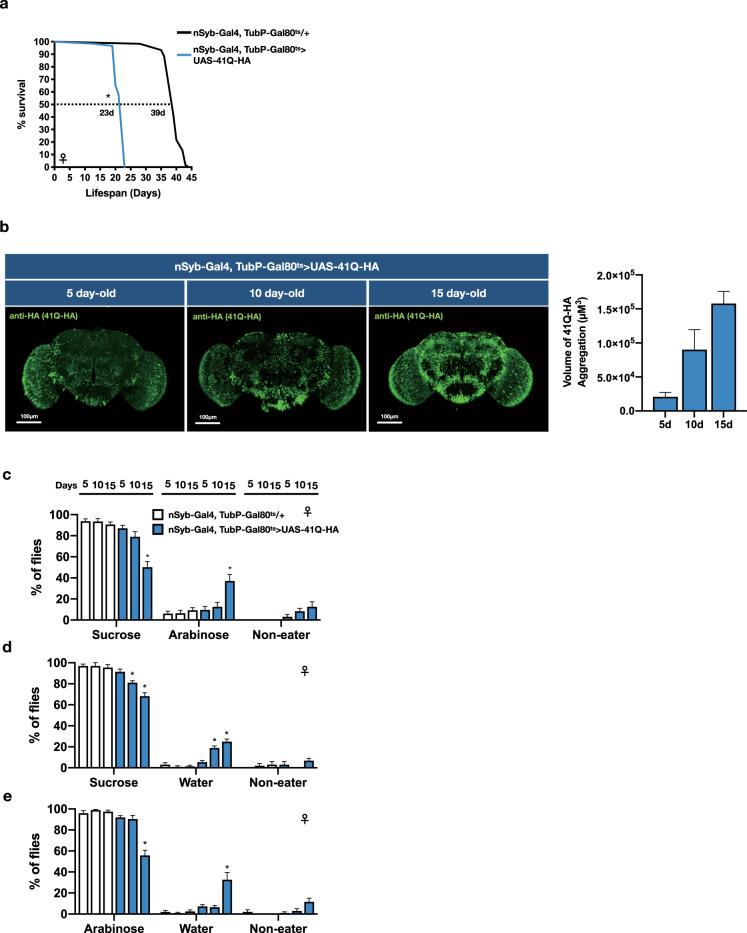
The efficacy of making proper VBFD is impaired in the polyQ-expressing flies. **a** Survival curves of flies with adult-onset ectopic expression of 41Q-HA in the nervous system. Results were analyzed by the log-rank test. *n* = 60 for each condition. *p < 0.01. **b** Brain images of female flies expressing 41Q-HA for 5, 10, and 15 days. Brain samples were stained with anti-HA (green, stained for 41Q-HA). Scale bars, 100 μm. The volume of 41Q-HA aggregates was quantified as described in the “Methods”. **c**–**e** VBFD assays were performed in female flies of different chronological ages (5, 10, and 15 days). The food choices used were 150 mM sucrose and 150 mM arabinose in (**c**); 150 mM sucrose and plain water in (**d**); 150 mM arabinose and plain water in (**e**). Results were expressed as means ± SEM and analyzed by two-way ANOVA. The statistical significance was assessed by the comparison to age-matched controls. *n* = 100 for each condition. *p < 0.01. Genotypes: control (TubP-Gal80^ts^/+; nSyb-Gal4/+); 41Q-HA-expressing flies (TubP-Gal80^ts^/+; nSyb-Gal4/UAS-41Q-HA). The above flies were developed at 18 °C and transferred to 29 °C right after eclosion. VBFD assays were performed at 29 °C.

Next we like to know whether VBFD is affected in the fly model of neurodegenerative disorders. In this study, the polyglutamine-(polyQ-)induced toxicity was used to promote neurodegeneration^16^. The 41Q-HA polypeptides, which contain the HA-tagged 41 polyglutamine residues, were expressed specifically in adult neurons using the TARGET expression system^17^. Survival analysis indicated the median lifespan of 41Q-expressing female flies was ~23 days, almost half of the control flies in 29 °C (Fig. 3a). Accordingly, VBFD assays were performed in 5-, 10-, and 15-day-old diseased female flies and the 41Q expression was verified by anti-HA staining (Fig. 3b). As expected, adult flies expressing 41Q for 5 and 10 days still exhibited proper complex VBFD as the polyQ aggregates were gradually accumulating (Fig. 3c). However, after the expression of 41Q for 15 days, only ~50% of the starved diseased flies chose the calorie-rich sucrose. The rest either only ingested the non-metabolizable arabinose or did not consume any food. For the efficacies of simple VBFD, as the expression of 41Q increased, more and more hungry diseased flies chose the non-nutritious water, suggesting the deterioration of simple VBFD and likely the impairment of sweet sensation (Fig. 3d, e). Besides the TAGET expression system, we also exploited the GeneSwitch (GS) system to induce the neuronal-specific expression of polyQ proteins in adult flies, thereby avoiding the influences of temperature shifts that were used in the TARGET system. The efficacy of VBFD in such flies was also significantly impaired (Supplementary Fig. 7a). Taken together, these results suggest the efficacy of proper VBFD is progressively deteriorating in polyQ flies. More importantly, additional to the classical associative learning and memory assays, which involve the pre-conditioning steps, the VBFD analysis could be adapted as an easy and robust assay to determine and quantitate the cognitive disability in diseased flies.

### Effects of LiCl to the polyQ-mediated impairment of VBFD

**Fig. 4:**
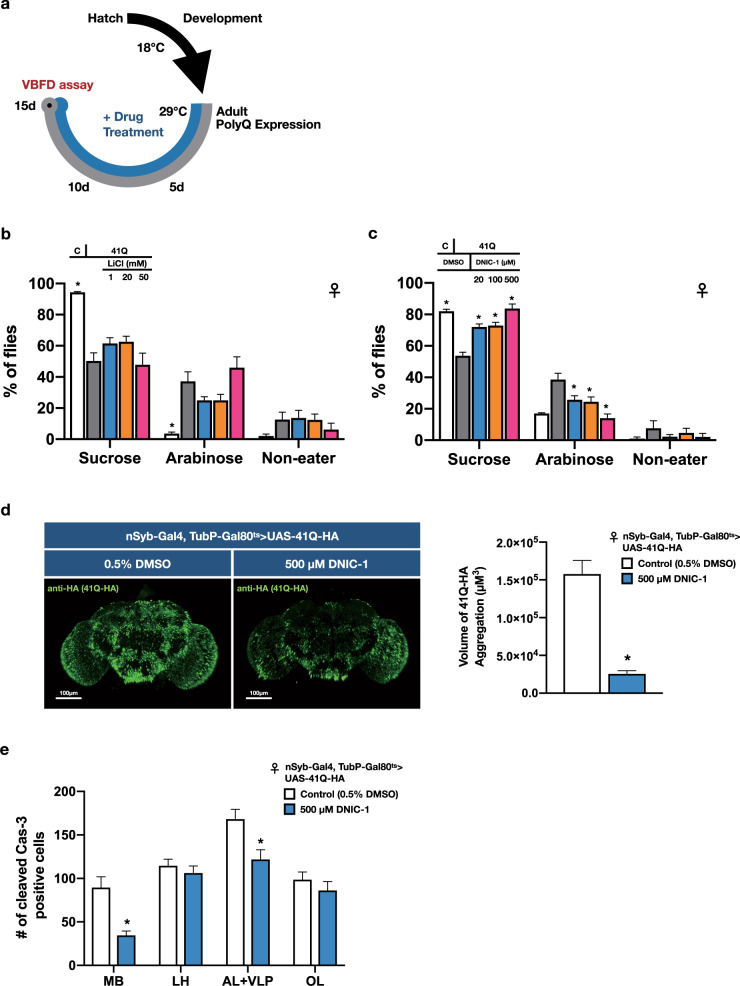
Identification of chemicals that ameliorate the polyQ-induced impairment of VBFD. **a** The experimental paradigms of 41Q-HA expression and drug treatments. **b**, **c** VBFD assays (150 mM sucrose vs. 150 mM arabinose) were performed in 15-day-old drug-treated and control female flies. The drugs used were 1, 20, and 50 mM LiCl in (**b**); 20, 100, and 500 μM DNIC-1 in (**c**). Results were expressed as means ± SEM and analyzed by two-way ANOVA. *n* = 100 for each condition. The statistical significance was assessed by the comparison to 41Q-HA-expressing flies fed with 0.5% DMSO. *p < 0.01. **d** Administration of 500 μM DNIC-1 for 15 days reduced the accumulation of 41Q-HA aggregates in adult brains. Brain samples were stained with anti-HA antibody (green; stained for 41Q-HA). Scale bars, 100 μm. The volume of 41Q-HA aggregates was quantified as described in the “Methods”. Results were expressed as means ± SEM and analyzed by Mann–Whitney test. *n* = 10 for each condition. *p < 0.01. **e** Administration of 500 μM DNIC-1 for 15 days markedly reduced the number of cleaved Cas-3-positive cells in the mushroom body region of 41Q-HA-expressing female flies. MB, mushroom body; LH, lateral horn; AL+VLP, antenna lobe and ventrolateral protocerebrum; OL, optic lobe. Results were expressed as means ± SEM and analyzed by Mann–Whitney test. *n* = 10 for each condition. *p < 0.01. Genotype for 41Q-HA-expressing flies: TubP-Gal80^ts^/+; nSyb-Gal4/UAS-41Q-HA. The above flies were developed at 18 °C and transferred to 29 °C right after eclosion. VBFD assays were performed at 29 °C.

A variety of compounds and genetic treatments have been shown to confer protective capability against polyQ-mediated pathologies in the *Drosophila* model^18^. The most visible pathological feature is the polyQ aggregates. Chemicals, such as histone deacetylase inhibitors^19^ and LiCl^20,21^, can reduce the polyQ aggregates in affected cells. In addition, these protective compounds also partly mitigate several deleterious phenotypes, including eye degeneration, decreased motor activity, and shortened lifespan^18^. Despite the therapeutic potential against distinct polyQ pathologies, the effectiveness of drug administration to alleviate the cognitive impairment has not been directly tested, given the lack of convenient assays. Here, the VBFD assay was used to determine the cognitive status of diseased flies treated with LiCl (Fig. 4a). Surprisingly, unlike its protective capability to most polyQ-induced defects examined in the earlier *Drosophila* studies, the impairment of complex VBFD was not alleviated by the treatment of LiCl (Fig. 4b). Application of either 1 or 20 mM of LiCl to 41Q-expressing flies only slightly increased the sucrose-ingesting population, while 50 mM LiCl had no effects. Intriguingly, simple VBFDs were partly rescued by the LiCl treatment, suggesting LiCl moderately attenuates the polyQ-mediated disruption of sweet sensation, but fails to improve the decline of complex VBFD (Supplementary Fig. 8a, b).

### A novel and steady NO-releasing dinitrosyl iron complex (DNIC-1) ameliorates the polyQ-mediated pathologies

In addition to LiCl, several studies have suggested the neuroprotective properties of nitric oxide (NO)^22,23^. To explore the protective functions of NO in the *Drosophila* model, a novel NO-releasing compound, dinitrosyl iron complex [(NO)_2_Fe(μ-SCH_2_CH_2_OH)_2_Fe(NO)_2_] (DNIC-1), was supplemented in the food^24–27^. As shown in Fig. 4c, DNIC-1 treatment effectively ameliorated the impairment of complex VBFD in 15-day-old female flies suffering from polyQ-mediated neurodegeneration. The fidelity of VBFD could be effectively maintained by the feeding of 500 μM DNIC-1. In addition, the feeding decision-related cognitive function was much less impaired in diseased flies treated with 20 and 100 μM DNIC-1, suggesting a dose-sensitive response (Fig. 4c). As anticipated, the impaired simple VBFD was almost completely preserved by the treatment of 500 μM DNIC-1 (Supplementary Fig. 9a, b). To ascertain the protective effects is NO-dependent, we found the degraded DNIC-1 (use after passing 10× half-lives) was not able to relieve the impairment of VBFD in 41Q flies (Supplementary Fig. 9g). Moreover, a stable radical scavenger for NO, PTIO, when co-administrated with DNIC-1, markedly abolished the protective function of DNIC-1, substantiating the involvement of NO (Supplementary Fig. 9g). Besides slowing down the deterioration of VBFD, DNIC-1 treatment also moderately extended the median lifespan of polyQ flies (Supplementary Fig. 9c; ~18% increase), and restored the eye degeneration and locomotor disability (Supplementary Fig. 9d and Supplementary Movie 1). Together, our results suggest ectopic NO is beneficial to distinct polyQ pathologies. In addition, to avoid the influences of genetic background, the neuroprotective effects of DNIC-1 were also validated in the polyQ flies generated via the GeneSwitch system. Treatment of DNIC-1 also delayed the impairment the VBFD in such flies (Supplementary Fig. 7a).

Next, to better understand the mechanisms of DNIC-1-mediated neuroprotection, we found levels of polyQ aggregates were greatly reduced by the DNIC-1 treatment (Fig. 4d and Supplementary Fig. 7b). The reduction of polyQ aggregates could result from enhanced proteolysis or decreased polyQ synthesis. However, the latter seems less likely given the expression levels of cellular factors, such as Elav and Repo, were not affected by the administration of DNIC-1 (Supplementary Fig. 9i). It is currently not clear what protein clearance pathways are affected by DNIC-1. Furthermore, in consistent with the reduction of polyQ aggregates, the number of brain cells undergoing cell death was notably decreased in DNIC-1-treated flies (Fig. 4e). Quantification of active Cas-3-positive cells in the selected brain regions indicated the DNIC-1-elicited protective effects were most prominent in the mushroom body (MB), which is a conserved brain structure that plays critical roles in diverse behaviors, including olfaction, associative learning, sleep, and feeding^28–30^. Therefore, it is possible that loss of MB neurons may interfere with the making of proper VBFD and lead to impaired cognition.

### Neural circuits that govern the efficacy of VBFD

**Fig. 5:**
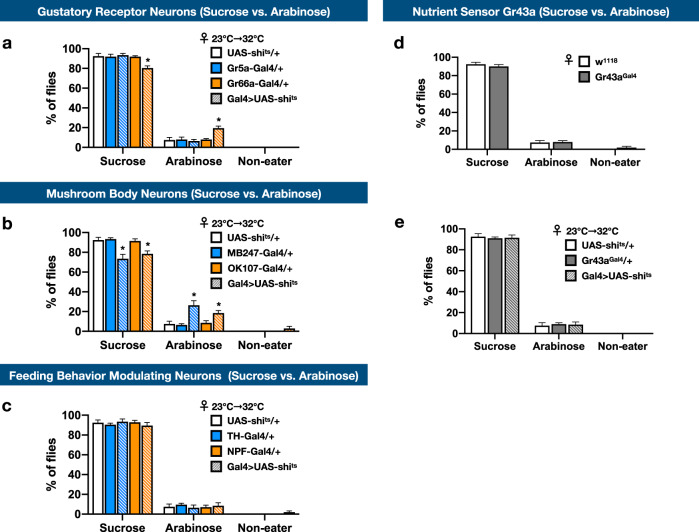
Identification of neural circuits that modulate the making of complex VBFD. **a**–**c**, **e** VBFD assays (150 mM sucrose vs. 150 mM arabinose) were performed in female flies that have reduced activity in (**a**) gustatory receptor neurons (Gr5a-Gal4 and Gr66a-Gal4), (**b**) mushroom body neurons (MB247-Gal4 and OK107-Gal4), (**c)** feeding behavior modulating neurons (TH-Gal4 and NPF-Gal4), and (**e)** Gr43a-positive neurons (Gr43a^Gal4^). **d** VBFD assays were performed in female Gr43a mutants (Gr43a^Gal4^) at 25 °C. Results were expressed as means ± SEM and analyzed by two-way ANOVA. *n* = 100 for each condition. Note all the columns of UAS-shi^ts^/+ were the same. In **a**–**c**, **e**, the flies were reared at 23 °C until 1 h before the VBFD assay (32 °C). The statistical significance was assessed by the comparison to UAS-shi^ts^/+ controls. In (**d**), the flies were reared at 25 °C and the VBFD assays were performed at 25 °C. The statistical significance was assessed by the comparison to w^1118^. *p < 0.01.

To locate the neural pathways that govern the making of VBFD, we altered the activity of known neuronal types related to the feeding behaviors, starting from the periphery to the central brain. Suppression of neuronal activity was mediated by the expression of Shibire^ts^ (Shi^ts^), a temperature-sensitive mutation of *Drosophila* dynamin, to block the synaptic transmission in targeted cell types. In consistent with earlier studies, the competence to read the nutritional values and make the proper VBFD was not affected by silencing the activity of gustatory receptor 5a (Gr5a)-positive sweet sensing neurons^3^ (Fig. 5a and Supplementary Fig. 10a, b). However, intriguingly, suppression of the Gr66a-positive bitter-sensing neurons slightly blunted the complex VBFD (Fig. 5a and Supplementary Fig. 10a, b). Therefore, as the making of proper VBFD may not require the activity of sweet neurons, the bitter neurons may partly modulate the efficacy of proper VBFD^3,31^. Next, in considering the MB neurons are implicated in diverse aspects of feeding behaviors, such as the integration of hunger/satiety signals and modulation of food-seeking^30^, the VBFD assay was performed in flies with altered MB activity. Silencing the activity of MB247- or OK107-labeled MB neurons led to moderate changes of VBFD. The sucrose-ingesting population was reduced to ~70%, comparing to ~90% of control 10-day-old young flies, suggesting the proper VBFD may be partly governed by MB neurons (Fig. 5b and Supplementary Fig. 10c, d). However, since the VBFD was not fully impaired when MB function was blocked, indicating additional brain regions may participate in VBFD making. However, it is still possible Shi^ts^ proteins may not completely silence the MB activity. Finally, we examined the VBFD in flies with altered dopaminergic rewarding system and feeding-promoting NPF signals^12,32,33^. Surprisingly, the making of proper VBFD remained efficient even when either pathway was perturbed, suggesting other central brain pathways may dictate the proper VBFD (Fig. 5c and Supplementary Fig. 10e, f). One plausible candidate is the known nutrient sensor in the *Drosophila* brain, Gr43a^9^. As shown in Fig. 5d, e (more in Supplementary Fig. 10g–j), the efficacy of complex VBFD was not affected by the loss of Gr43a receptors or inhibition of Gr43-positive neurons. Since its functional role in sensing the levels of hemolymph fructose and modulating food consumption, the inability of Gr43a-linked circuits to modulate the efficacy of VBFD further indicates the making of VBFD may depend on multiplexing central brain circuits. Together, our results suggest making proper VBFD may require sophisticated computing processes in the nervous system and MB neurons are a part of the circuits.

## Discussion

Earlier studies have indicated starved fruit flies are able to evaluate and learn the nutritional value of sugar solutions^3,6–8^. Particularly, making such VBFD is robust and a native behavior. Consistently we noticed the flies defective of learning and memory, such as Rut and Dnc mutants, could still made proper VBFD (Supplementary Fig. 10k–m). More interestingly, recent studies also indicate the capability of fruit flies to detect nutritional values of sugars involves a taste-independent metabolic-sensing pathway^3,8^. But how this metabolic-sensing pathway finalizes the feeding decision is far from clear. Decision-making is an important feature that allows every living animal to identify and choose alternatives based on various criteria, therefore representing a unique spectrum of cognition. Food-deprived flies, once encounter the potential foods, must quickly evaluate the qualities of foods and select the food choice with nutritional value to resolve the energy need. Consequently, the feeding decision based on the caloric contents of foods needs to be properly made and maintained. In this study, we deployed the VBFD assay to determine the cognitive status of *Drosophila*. Perceivably, the feeding decision between the metabolizable sucrose and non-metabolizable arabinose is more difficult to consolidate than the decision between sweet sugar and plain water, given the differentiation of nutritional values of sugar solutions should be more complicated than the identification of palatable sugar. Along these lines, the VBFD assay and the combinations of testing food substances allowed us to distinguish two distinct levels of cognitive function in the forms of complex and simple VBFDs. By monitoring the efficacy of VBFD, our results showed the deterioration of complex VBFD is more severe in aged flies, while the simple VBFD and perhaps the basic sweet sensation remain less affected (Fig. 1b–d). Therefore, it appears the decline of complex cognitive functions is more sensitive to the aging process. Moreover, based on the experimental specifications, the complexity levels of feeding decision can be directly characterized.

As demonstrated by the VBFD assay, the aged fruit flies start to made improper feeding decisions by intaking only the non-metabolizable arabinose solution or becoming the non-eaters (e.g., Fig. 1b). Despite the sweet sensations in aged flies are still functional (Fig. 1c, d) and most aged flies can initiate food intake in response to the hungry signals (Supplementary Fig. 2), is it possible that the duration of starvation impacts the efficacy of making proper VBFD? As shown in Supplementary Fig. 11a, b, the glycogen/glucose levels in the 10-day-old young flies were reduced to about half of the control condition after 12 h of starvation and reduced to about one-third after 24 h of starvation. Therefore, it is clear that the durations of starvation can differentially affect the levels of glycogen/glucose in adult flies. However, judging from the results of VBFD assays shown in Supplementary Fig. 11c–e, the efficacies of making proper VBFD were not disturbed in the 10-day-old young flies with lower levels of glycogen/glucose (either 12 or 24 h of starvation), suggesting the states of energy reserves may only have little impact on the processes of VBFD making. Moreover, under the normal feeding condition (no starvation), the aged flies (60-day-old) exhibited lower glycogen/glucose levels than the young fruit flies (10-day-old) (Supplementary Fig. 11a, b). After starving the aged flies for 12 h, the glycogen/glucose levels were further reduced. Interestingly, the levels of glycogen/glucose in aged flies that had been food deprived for 12 h were similar to the young flies starved for 24 h (Supplementary Fig. 11a, b), suggesting their states of energy reserves are comparable. However, the efficacy of making proper VBFD was deteriorating in the 60-day-old aged flies (e.g., Fig. 1b), suggesting the age-dependent changes of energy reserves may not be the main factor mediating the impairment of VBFD. Instead, the functional integrity of neural circuits governing the feeding decisions may be the key modulator that determines the efficacy of VBFD.

There are a number of genetic manipulations and chemicals that have pro-longevity effects in the Drosophila model^14,15,34,35^. However, in addition to the extended life expectancy and improved motor performance in aged flies, their effects to the age-dependent cognition decline have not been systematically explored. In this study, our results showed the efficacies of both complex and simple VBFDs are significantly preserved in the Hsp22-expressing and LiCl-treated long-lived aged flies (Fig. 2b–e), suggesting, at least in these two cases, the pro-longevity treatments are able to delay the deterioration of feeding-related cognitive behaviors. However, unlike our results, a previous study has shown the efficacy of olfaction-associated learning and memory is not retained in the dietary-restricted long-lived flies^36^. Since each pro-longevity treatment may exert its effects through distinct mechanisms, it is not clear if other pro-longevity treatments have similar protective effects to the age-dependent cognitive decline. More interestingly, it has been demonstrated in several studies that tissue-specific expression of selected pro-longevity gene is sufficient to prolong the animal’s lifespan^14,35,37,38^. In that case, learning how these long-lived flies perform in the VBFD assay will allow us to substantiate and characterize the functional inter-connections between the peripheral tissue/organ and the central nervous system, such as the gut–brain communications^10,38,39^.

Cognitive impairment is frequently associated with neurodegenerative diseases. In consistent with this notion, the efficacies of both complex and simple VBFDs were drastically jeopardized in ployQ flies, indicating the development of cognitive disorders and the loss of sweet sensation (Fig. 3c–e). Unfortunately, the available treatments for neurodegenerative diseases only manage the symptoms or slow down the disease progression. It is also extremely difficult to improve the cognitive impairment associated with neurodegenerative diseases. The neuroprotective effects of LiCl have been demonstrated in several recent studies, including in the SCA3 *Drosophila* model, which is also a polyQ neurodegenerative disorder^18,20,21^. Chronic administration of LiCl alleviates SCA3-mediated pathologies, such as eye degeneration, locomotor disability, and shortened lifespan. Despite its neuroprotective potential, however, the effect of LiCl to relieve the impairment of cognition has never been examined. Unexpectedly, our VBFD analysis indicated LiCl treatment has only limited effects on the polyQ-mediated impairment of complex VBFD, suggesting the complex cognitive behaviors are more sensitive to the degenerative pathology (Fig. 4b and Supplementary Fig. 8). Regarding the neuroprotective properties of NO, in this study, we found DNIC-1, a novel NO-releasing chemical, is a promising neuroprotective compound that mitigates diverse polyQ-dependent phenotypes, including the decline of cognitive function (Fig. 4c–e and Supplementary Fig. 9). However, we also noted the protective effects of DNIC-1 is most profound when the compound is administrated along with, but not after, the onset of 41Q expression (Supplementary Fig. 9e–f). In considering the unstable nature and short half-life of NO (~sec–min), selection/development of the prodrug featuring steady and long-term NO-releasing ability is a critical step for the translation/development of NO as a novel therapeutic agent, especially for chronic diseases. At present, there are several classes of NO donor chemical available. In addition to FDA-approved organic nitrates (i.e. glycerol trinitrate), the NONOate variants of NO donor chemical release NO through a hydrolytic mechanism and have distinct half-life (*t*_1/2_ = 1 min to 20 h at pH 7.4, 22–25 °C). The design of DNIC-1 [(NO)_2_Fe(μ-SCH_2_CH_2_OH)Fe(NO)_2_], a novel NO-delivery reagent used in this study, is based on a natural motif, dinitrosyl iron unit [Fe(NO)_2_] for the delivery and storage of NO^23–26^. In comparison to the burst release of NO by the FDA-approved organic nitrates and well-studied NONOates, DNIC-1 displays a steady kinetics for O_2_-triggered release of NO (half-life = 27.4 h at pH 7.4, 22–25 °C), providing a more stable source of ectopic NO under physiological conditions. To verify the neuroprotective effects is mediated by NO, the degraded DNIC-1 was not able to save the impaired VBFD (Supplementary Fig. 9g). Moreover, feeding of the NO scavenger, PTIO, markedly accelerated the deterioration of VBFD elicited by polyQ aggregates (Supplementary Fig. 9h). Based on the levels of polyQ aggregates, DNIC-1 treatment was able to effectively reduce the aggregations of polyQ polypeptides and down-regulate the cell death events in the affected brains (Fig. 4d, e). There are several possible mechanisms, such as activation of abnormal protein clearance machinery, reduction of disease gene expression, and modulation of cellular factors related to cell death/survival^18,40,41^. Based on our earlier analyses, the levels of glycogen/glucose, which may reflect the current state of energy reserves, were not markedly changed by the DNIC-1 treatment (Supplementary Fig. 9j). Therefore, the above possibilities may need to be validated by additional studies. Finally, given its capability to activate the clearance of disease protein aggregates, it will be worth exploring the protective capability of DNIC-1 in other neurodegenerative diseases and in higher animal models.

## Methods

### Fly stocks

Flies were reared on regular cornmeal diet and housed in standard conditions. The following laboratory fly lines were used: TubP-Gal4, nSyb-Gal4, OK107-Gal4, MB247-Gal4, TubP-Gal80^ts^; nSyb-Gal4. We obtained the following fly lines from Bloomington Drosophila Stock Center: w^1118^ (BDSC 3605), NPF-Gal4 (BDSC 25681), TH-Gal4 (BDSC 8848), Gr5a-Gal4 (BDSC 57591), Gr66a-Gal4 (BDSC 57670), UAS-shi^ts^ (BDSC 44222), Rut^2080^ (BDSC 9405), Dnc^1^ (BDSC 6020), UAS-HTT128Q^FL^ (BDSC 33808), and UAS-miR-rCD2^42,43^. Gr43a^Gal4^ was obtained from Dr. Hubert Amrein, Texas A&M University^9^. GMR-Gal4, UAS-41Q-HA, and UAS-41Q-HA were obtained from Dr. Horng-Dar Wang, National Tsing Hua University, Taiwan^44^. Elav-GeneSwitch (ElavGS) was obtained from Dr. Chun-Hong Chen, National Health Research Institutes. In most cases, flies were reared at 25 °C. For the Gal4/UAS-shi^ts^ experiments, flies were reared at 23 °C until 1 h before the VBFD assay (32 °C). To express UAS-41Q-HA in adult flies via the TARGET system^17^, animals were allowed to develop at 18 °C and transferred to 29 °C right after eclosion.

### VBFD assay (based on two-choice feeding assay)

The two-choice feeding assay was performed according to previous studies with minor modifications^3–5^. Briefly, 20 flies were food-deprived on 1% agar at 12 a.m. for 12 h. At 12 p.m., flies were mildly anesthetized by CO_2_ and quickly introduced to a 10 cm Petri dish that contains two different liquid substances. Total eight droplets (four droplets for each food choice) were evenly placed around the Petri dish. Each liquid droplet contains 20 μL solution. The solution was either color labeled with 0.01% erioglaucine disodium salt (blue dye; Acros Organics, Cat# 229730250) or 0.1% Food Red No. 106 (red dye; TCI, Cat# F0143). The dye added was randomly chosen in each round of experiments. In most cases, feeding assays were performed in complete darkness for 2 h at 25 °C. For the 41Q-HA-expressing flies, feeding assays were conducted at 29 °C. For Gal4/shi^ts^ flies, the feeding assays were conducted at 32 °C. The feeding decision of fruit flies was observed under the stereomicroscope and scored by color (B: blue [ingesting only the blue food], R: red [ingesting only the red food], P: purple [ingesting both blue and red foods], and N: non-eater [no color accumulation in the abdomen]) accumulated in the abdomen. The results were presented as percentages of (1) (#_B_ + 1/2#_P_)/#_total_ (number of flies tested), (2) (#_R_ + 1/2#_P_)/#_total_, and (3) #_N_/#_total_. Two sugars were used in the assay: sucrose (Acros Organics, Cat# 177142500) and arabinose (Alfa Aesar, Cat# A10357).

### Generation of UAS-hsp22-HA transgenic line

Total RNA (TRIzol reagent from Sigma, Cat# T9424) from the heads of 20 female w^1118^ flies was used to generate the *Drosophila* cDNA library (HiScript I First Strand cDNA Synthesis KIT from BIONOVAS, Cat# AM0675-0050). Subsequently, DNA fragments containing the hsp22-HA were PCR amplified from the cDNA library and cloned into the *Eco*RI and *Xho*l sites of pUASTattB (GenBank: EF362409.1). Sequences of the DNA insert were verified before transgenesis. The UAS-hsp22-HA was inserted to the attP40 site on the second chromosome for the creation of transgenic line (WellGenetics, Taiwan). Primers used are listed in Supplementary Table 1.

### Generation of UAS-miR-hsp22 transgenic line

The functional stem–loop structure of the artificial mir-based RNAi_Hsp22 miRNA was created through the first primer set_ dme-Hsp22-mir-1 or dme-Hsp22-mir-2 primers by PCR reaction (Supplementary Table 1). This functional stem–loop miRNA was then extended and added flanking sequences with restriction enzyme sites by the second primer set_ Mir6.1_5′*Eco*RI/*Bgl*II and Mir6.1_3′*Not*I/*Bam*HI primers to get precursor Hsp22 miRNA unit. The *Bgl*II and *Bam*HI restriction enzyme sites of precursor Hsp22 miRNA unit were used for assembling of multiple copies to generate the Hsp22-2miRNAs cassette. Finally, the restriction enzyme double-digested *Eco*RI/*Bam*HI-Hsp22-mir1 and *Bgl*II/*Not*I-Hsp22-mir2 were concurrently integrated into the *Eco*RI and *Not*I site region of pUASTattB vector (GenBank: EF362409.1) to generate the pUASTattB_miR-Hsp22-2miR plasmid^45^. The UAS-mir-hsp22 was inserted to the attP40 site on the second chromosome for the creation of transgenic line (WellGenetics, Taiwan). Primers used are listed in Supplementary Table 1.

### Drug treatment

Briefly, for the drug treatment, flies were raised on the regular cornmeal diet containing the selected drug(s). LiCl (Acros Organics, Cat# 199881000) was dissolved in ddH_2_O and diluted to indicated concentrations in the regular fly food. DNIC-1 and PTIO (Sigma, Cat# P5084) were dissolved in DMSO (Acros Organics, Cat# 348441000) and diluted to final concentration(s) in the food. The final concentration of DMSO was kept at 0.5% in all cases. DNIC-1 was synthesized according to previous publications^24–27,46^.

### Lifespan assay

Unmated flies were collected and separated by gender. Twenty flies were housed in a vial. In most of our experiments, flies were incubated at 25 °C, except for the flies used for adult-specific expression. Flies carrying the TARGET genetic elements were reared at 18 °C until eclosion and transferred to 29 °C. Every 2–3 days, flies were transferred to a new vial containing fresh medium and number of the dead flies was counted and recorded. Survival analyses were performed using Prism 7 (GraphPad Software). To verify the life-supporting capability of sucrose and arabinose, 1% agar containing 150 mM of selected sugar was used as the sole food source of 1-day-old w1118 flies. Flies were transferred to a new vial containing fresh food preparation every 12 h, and their survival was monitored and recorded.

### *Drosophila* eye morphology

Ten-day-old flies of indicated genotypes were anesthetized by CO_2_ and the eye images were captured under the stereomicroscope.

### Immunohistochemistry

Fly brains were dissected and fixed by 4% paraformaldehyde (Electron Microscopy Sciences Cat# 15710) for 25 min at room temperature. The brain samples were washed three times with PBST (1× PBS and 0.5% Triton X-100) and incubated in PBST overnight at 4 °C. Subsequently, the brain samples were incubated with primary antibodies (diluted in PBST plus 5% of normal goat serum) overnight at 4 °C. Next, brain samples were washed three times with PBST and incubated with secondary antibodies for another night at 4 °C. Finally, the brain samples were mounted using the SlowFade Gold antifade reagent (Invitrogen, Cat# S36936). Brain images were captured by the Leica SP8 confocal microscope system. Imaris (ver 9.1, Bitplane) was used to process and analyze brain images. Primary antibodies used in this study were: rat anti-HA (1:250; Roche 3F10, RRID: AB_2314622), rabbit anti-cleaved Caspase-3 (1:200; Cell Signaling Technology 9661, RRID: AB_2341188), rat anti-Elav (1:100; DSHB, Elav 7E8A10, RRID:AB_528218), and mouse anti-Repo (1:75; DSHB 8D12, RRID:AB_528448). Secondary antibodies used in this study were goat anti-rat Alexa 488 (1:500; Invitrogen, A11006), goat anti-rabbit Alexa 568 (1:500; Life Technologies, A11036), and goat anti-mouse Alexa 647 (1:500; Life Technologies, A21236).

### Quantification of glycogen and glucose levels

Three flies were homogenized in 200 µL PBS; the homogenates were then boiled for 10 min to inactive enzymes. The boiled samples were then centrifuged for 10 min at 4 °C at 15,000 × *g* to remove insoluble materials. One microliter of supernatant was used for glycogen quantification by using the Glycogen Assay kit (Abcam; ab65620). Twenty microliters of supernatant was used for glucose quantification by using Glucose Assay kit (Randox; GL2623). Three independent replicates (*n* = 9) per condition were used for both assays.

### Quantification of sucrose intake

Briefly, ten flies were placed in a vial with 1% agar containing 150 mM sucrose and 0.01% erioglaucine disodium salt (blue dye; Acros Organics, Cat# 229730250). After feeding for 2 h, five flies were homogenized in 1000 µL PBS. The homogenates were then filtered through a 0.45 µm syringe filter. The 625 nm absorbance was recorded by using a spectrophotometer. Five independent replicates (*n* = 25) per condition were used.

### Quantification and statistical analysis

Statistical tests were conducted using Prism 7 (GraphPad Software). Results of feeding decision were expressed as means ± SEM and analyzed by two-way ANOVA with multiple comparisons (Dunnett’s test). The survival curves were plotted as Kaplan Meyer plots and the statistical significance was tested using the log-rank test. Quantification of 41Q-HA aggregates in the brain images was performed using the “Surface Function” of Imaris. The brain region used for the quantification is outlined in Supplementary Fig. 12a. Number of cleaved Caspase-3-positive cells was calculated using the “Spots Function” of Imaris. Supplementary Figure 12b outlined the brain regions used to measure the dying brain cells (MB, mushroom body; LH, lateral horn; AL+VLP, antenna lobe and ventrolateral protocerebrum; OL, optic lobe). The quantitative values were expressed as means ± SEM and analyzed by Mann–Whitney test. All the asterisks in statistical tests indicate statistical significance compared to the corresponding controls (*p < 0.01).

### Reporting summary

Further information on research design is available in the Nature Research Reporting Summary linked to this article.

## Supplementary information

Supplementary Information

Supplementary Movie 1

Reporting Summary

## Data Availability

All data are available in the manuscript or the supplementary materials; raw data are available upon request.
