# Supplementary material for: Assessing the cognitive status of Drosophila by the value-based feeding decision
Source: NPJ Aging Mech Dis. 2021 Sep 15;7:24. doi: 10.1038/s41514-021-00075-6 (PMC8443761; doi:10.1038/s41514-021-00075-6)
Supplement: Supplementary file 1 — Supplementary Information [file 41514_2021_75_MOESM1_ESM.pdf]

Supplementary Information for

Assessing the Cognitive Status of Drosophila by the Value-Based Feeding Decision

Chih-Chieh Yu[1,2], Ferng-Chang Chang[2,3], Yong-Huei Hong[4], Jian-Chiuan Li[5], Po-Lin Chen[5], Chun-Hong Chen[5], Tzai-Wen Chiu[2,3], Tsai-Te Lu[4], Yun-Ming Wang[1,2], Chih-Fei Kao[2,3,*]
Correspondence to: kcfei@nctu.edu.tw

This PDF file includes:

Supplementary Figures 1 to 12
Caption for Supplementary Table 1
Caption for Supplementary Movie 1

Other Supplementary Information for this manuscript include the following:

Supplementary Table 1
Supplementary Movie 1

Supplementary Figure Legends

Supplementary Fig. 1. The efficacy of making proper VBFD in the aging flies

(a) Survival curves of w^{1118} flies fed on 1% agar (control) and 1% agar plus indicated sugar type (150mM sucrose or 150mM arabinose). n=40 for each condition. **(b)** Survival curves of w^{1118} flies. Median lifespan: female (orange), 63 days, n=97; Male (blue), 68 days, n=96. **(c-e)** VBFD assays were performed in male flies of different chronological ages (10, 40, and 60 days). The food choices used were 150 mM sucrose and 150 mM arabinose in **(c)**; 150 mM sucrose and plain water in **(d)**; 150 mM arabinose and plain water in **(e)**. Results of VBFD were expressed as means ±SEM and analyzed by two-way ANOVA with multiple comparisons (Dunnett's test). n=100 for each condition. *: $p<0.01$. Results of 40 day- and 60 day-old flies were compared to 10 day-old flies. All flies used in above assays were maintained at 25°C.

Supplementary Fig. 2. Flies consumed more sucrose solution after 12 hours starvation

(**a**) The abdomen images of control (no starvation; regular feeding) and starved flies (12 hours of starvation) feeding on 150 mM sucrose solution (blue dye labeled) for 2 hours. 10 day- and 60 day-old w^{1118} flies were used to measure the intake of sucrose solution. (**b**) Quantification of the sucrose intake in 2 hours. n=25 for each column. The results were analyzed by Mann-Whitney test. *: $p<0.01$. The statistical significance was assessed by the comparison to age-matched controls. All flies used in above assays were maintained at 25°C.

Supplementary Fig. 3. VBFD analyses in the Canton-S flies

(**a**) Survival curves of the Canton-S flies. n=60 for both genders. (**b-d**) VBFD assays were performed in female flies of different chronological ages: 10 and 60 days. The food choices used were 150 mM sucrose and 150 mM arabinose in (**b**); 150 mM sucrose and plain water in (**c**); 150 mM arabinose and

plain water in (**d**). Results were expressed as means ±SEM and analyzed by two-way ANOVA. n=100 for each condition. *: p<0.01. The statistical significance was assessed by the comparison to 10 day-old flies. All flies used in above assays were maintained at 25°C.

Supplementary Fig. 4. Aged long-lived flies still preserve the proper VBFD

(a-c) Results of VBFD assays for 90 day-old female flies expressing ectopic Hsp22. **(d)** Survival curves of Hsp22 over-expressing (OE; UAS-hsp22-HA) and knock-down (KD; UAS-miR-hsp22) male flies. Results were analyzed by the log-rank test. n=100 for each genotype. *: p<0.01. **(e-g)** VBFD assays were performed in Hsp22 OE and KD male flies of different chronological ages (10, 40, and 70 days). The food choices used were 150 mM sucrose and 150 mM arabinose in **(a)** and **(e)**; 150 mM sucrose and plain water in **(b)** and **(f)**; 150 mM arabinose and plain water in **(c)** and **(g)**. Results of all VBFD were expressed as means ±SEM and analyzed by two-way ANOVA with multiple comparisons (Dunnett's test). n=100 for each condition. *: p<0.01. The statistical significance was assessed by the comparison to age-matched controls. Genotypes: control (TubP-Gal4/+ and UAS-miR-rCD2/+; TubP-GAL4/+); OE (UAS-hsp22-HA/+; TubP-Gal4/+); KD (UAS-miR-hsp22/+; TubP-Gal4/+). All flies used in above assays were maintained at 25°C.

Supplementary Fig. 5. Neuronal-specific expression of Hsp22 maintains the proper VBFD during aging

(**a-b**) Survival curves of flies that express Hsp22 specifically in neurons (nSyb-GAL4>UAS-hsp22-HA). Genotype of the control flies was nSyb-GAL4/+. Results were analyzed by the log-rank test. n=100 for each genotype. *: p<0.01. (**c-e**) VBFD assays were performed in female flies with neuronal specific expression of Hsp22. Flies of two chronological ages were used: 10 and 70 days. The food choices used were 150 mM sucrose and 150 mM arabinose in (**c**); 150 mM sucrose and plain water in (**d**); 150

mM arabinose and plain water in (**e**). Results were expressed as means ±SEM and analyzed by two-way ANOVA. n=100 for each condition. *: p<0.01. The statistical significance was assessed by the comparison to age-matched controls. Genotype: control (UAS-hsp22-HA/+; +/+); OE: (UAS-hsp22-HA/+; nSyb-Gal4/+). All flies used in above assays were maintained at 25°C.

Supplementary Fig. 6. Survival analysis of LiCl-treated flies

Survival curves of w^{1118} flies fed with 1 mM LiCl. n=60 for both conditions. All flies used in the above assay were maintained at 25°C.

Supplementary Fig. 7. PolyQ flies generated using the GeneSwitch (GS) system

(**a**) VBFD assays (150 mM sucrose vs. 150 mM arabinose) were performed in 15 day-old drug-treated and control female flies. Results were expressed as means ±SEM and analyzed by two-way ANOVA. n=100 for each condition. *: p<0.01. Genotypes: elavGS/UAS-41Q-HA. (**b**) Brain images of female flies expressing 41Q-HA by elavGS, (**a**) no induction, (**b**) induction by RU486, and (**c**) induction by RU486 plus 500 μM DNIC-1. 41Q-HA aggregates were significantly reduced after 15 days of DNIC-1 administration. Anti-HA antibody was used to visualize 41Q aggregates (green; stained for 41Q-HA). Scale bars, 100 μm. All flies used in above assays were maintained at 25°C.

Supplementary Fig. 8. LiCl moderately improves the impairment of simple VBFD caused by polyQ expression

(a) and **(b)** VBFD results of 41Q-HA-expressing flies (sucrose vs. water and arabinose vs. water) fed with 1, 20, and 50 mM LiCl. Results were expressed as means ±SEM and analyzed by two-way ANOVA with multiple comparisons (Dunnett's test). The significance of differences was compared to

41Q-HA-expressing flies without LiCl treatment (gray-colored bars). n=100 for each condition. *: p<0.01. Genotypes: control (TubP-Gal80^{ts}/+; nSyb-Gal4/+); 41Q-HA-expressing flies (TubP-Gal80^{ts}/+; nSyb-Gal4/UAS-41Q-HA). The above flies were developed at 18°C and transferred to 29°C right after eclosion. VBFD assays were performed at 29°C.

Supplementary Fig. 9. DNIC-1 treatment provides protection to the polyQ-mediated pathologies

(a) and **(b)** VBFD results of 41Q-HA-expressing flies (sucrose vs. water and arabinose vs. water) fed with 20, 100 and 500 μM DNIC-1. **(c)** Survival curves of 41Q-HA-expressing female flies fed with 20, 100, and 500 μM DNIC-1. **(d)** Eye images of 41Q-HA-expressing flies fed with 0.5% DMSO and 100 μM DNIC-1. **(e)** The experimental paradigms of 41Q-HA expression and DNIC-1 treatment. In this paradigm, three time points of drug treatment were indicated (total treatment days are 5, 10, 15 days, respectively). Please also note the duration (15 days) of 41Q-HA expression were kept the same for all conditions. Results of VBFD assays after the DNIC-1 treatment were shown in **(f)**. **(g)** VBFD assays were performed in 41Q-HA-expressing flies fed with 500 μM decayed DNIC-1 (after passing 10x half-lives) and 500 μM DNIC-1 + 500 μM PTIO (NO scavenger) for 15 days. **(h)** Results of VBFD assays on 41Q-HA-expressing flies fed with 500 μM PTIO for 10 days. Note that the columns of control flies fed with 0.5% DMSO and 41Q-HA-expressing flies fed with 0.5% DMSO and 500 μM DNIC-1 were the same as presented in Fig. 4c. Results of all VBFD assays shown above were expressed as means ± SEM and analyzed by two-way ANOVA with multiple comparisons (Dunnett's test). n=100 for each condition. The statistical significance was assessed by the comparison to respective controls. *: p<0.01. Genotypes: control (TubP-Gal80^{ts}/+; nSyb-Gal4/+); 41Q-HA-expressing flies (TubP-Gal80^{ts}/+; nSyb-Gal4/UAS-41Q-HA). **(i)** Immunostaining signals of Elav and Repo in the brain samples derived from control flies treated with 0.5% DMSO or 500 μM DNIC-1. n=5 for each condition. Quantifications of signal intensity were expressed as means ±SEM, and analyzed by Mann-Whitney test. Genotype:

TubP-Gal80^{ts}/+; nSyb-Gal4/+. Scale bar: 10 μM. The experimental flies were developed at 18°C except for the flies in (D) were at 25°C. After eclosion, flies were transferred to 29°C. VBFD assay were performed at 29°C. (**j**) The levels of glycogen and glucose from whole fly lysate were shown. Control: 15 day-old w^{1118} flies. DNIC: w^{1118} flies treated with 500 μM DNIC-1 for 15 days. The flies were reared at 29°C. n=9 for each column.

Supplementary Fig. 10. Activity of mushroom body is involved in making the complex VBFD

(a-f, h-j) VBFD assays (sucrose vs. water and arabinose vs. water) were performed in female flies that have reduced activity in **(a)** and **(b)** gustatory receptor neurons (Gr5a-Gal4 and Gr66a-Gal4); **(c)** and **(d)** mushroom body neurons (MB247-Gal4 and OK107-Gal4); **(e)** and **(f)** feeding behavior modulating neurons (TH-Gal4 and NPF-Gal4); and **(h)** and **(j)** Gr43a-positive neurons (Gr43a-Gal4). **(g-i, k-m)** VBFD assays were performed in female Gr43a^{Gal4} mutants **(g-i)**, Dnc, and Rut **(k-m)** mutants. 7~10-day old Gal4>UAS-shi^{ts} flies were used. 10 day-old Gr43a^{Gal4}, Dnc, and Rut female mutants were assayed. Results of all VBFD were expressed as means ±SEM and analyzed by two-way ANOVA with multiple comparisons (Dunnett's test). n=100 for each condition. Note that the columns of w^{1118} and UAS-shi^{ts}/+ in Figs. 5 and S5 were the same. In **(a-f, h-j),** the flies were reared at 23°C until one hour before VBFD assay (32°C). the statistical significance was assessed by the comparison to UAS-shi^{ts}/+ controls. In **(g-i, k-m),** the flies were reared at 25°C and VBFD assays were performed at 25°C. The statistical significance was assessed by the comparison to w^{1118}. *: $p<0.01$.

Supplementary Fig. 11. Levels of glycogen/glucose in fruit flies starved for 12 and 24 hours

(**a-b**) The levels of glycogen (a) and glucose (b) from the whole fly lysate of indicated condition. n=9 for each column. (**c-e**) Extending the starvation time (12 hours or 24 hours) had no impact on the efficacies of complex and simple VBFD. These assays were performed on 10 day-old flies. n=40 for

each column. Results were expressed as means ±SEM and analyzed by two-way ANOVA. n=40 for each condition. The statistical significance was assessed by the comparison to 12 hours starvation controls. Genotype: w^{1118}. All flies used in above assays were maintained at 25°C.

Supplementary Fig. 12. Schematic illustration of the brain regions analyzed

(a) and **(b)** The brain regions in the black outlines denoted the area where **(a)** 41Q-HA aggregation and **(b)** cleaved Caspase-3-positive cells were analyzed. **(a)** and **(b:** LH, AL+VLP, and OL**)** were analyzed with whole brain sections (1 µm/section) . **(b:** MB) was analyzed with posterior 1/2 sections. MB, mushroom body; LH, lateral horn; AL+VLP, antenna lobe and ventrolateral protocerebrum; OL, optic lobe.

Supplementary Table 1. List of primer sequences used for generating hsp22-HA and miR-hsp22 lines

Supplementary Mov. 1. DNIC-1 alleviates the polyQ-mediated motor dysfunction

This video showed the DNIC-1-treated polyQ-expressing flies have better motor activities. The animals used were 23 day-old female flies. At this age, untreated polyQ-flies already displayed strong motor defects. Left, 0.5% DMSO (controls). Right, 500 µM DNIC-1. Genotype for the 41Q-HA-expressing flies: TubP-$Gal80^{ts}$/+; nSyb-Gal4/UAS-41Q-HA.

Supplementary Figure 1.

1a

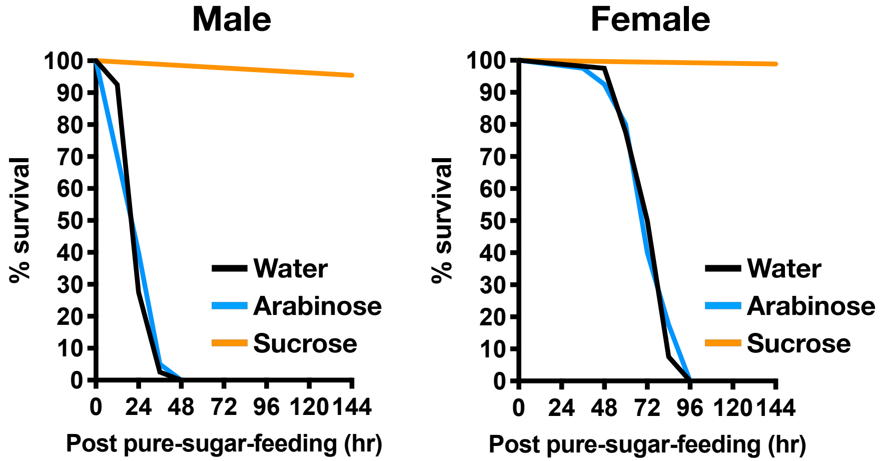

	Median Lifespan (hours)		Maximun Lifespan (hours)	
	Male	Female	Male	Female
Water	24	78	48	96
150mM Sucrose	>144	>144	>144	>144
150mM Arabinose	24	72	48	96

1b

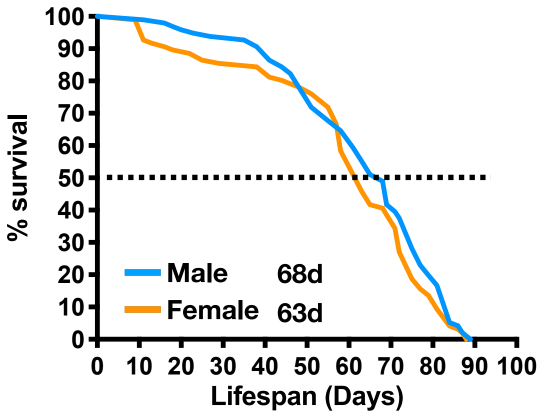

1c

1d

1e

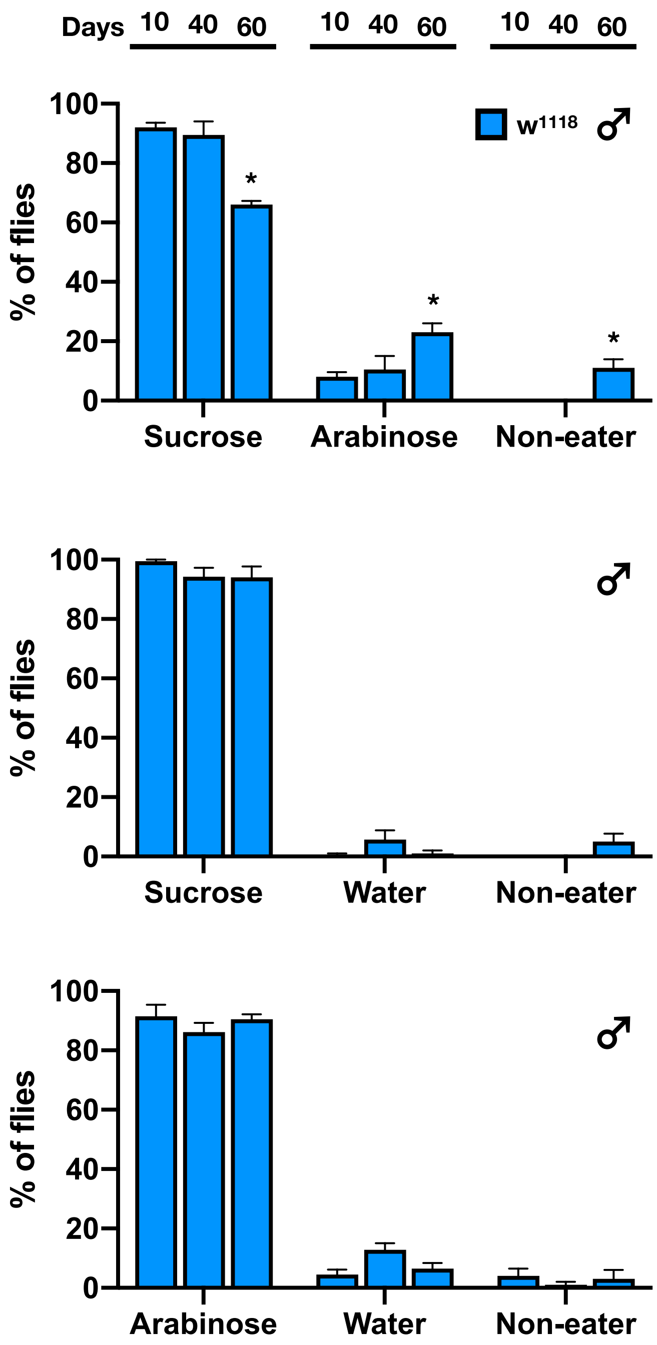

Supplementary Figure 2.

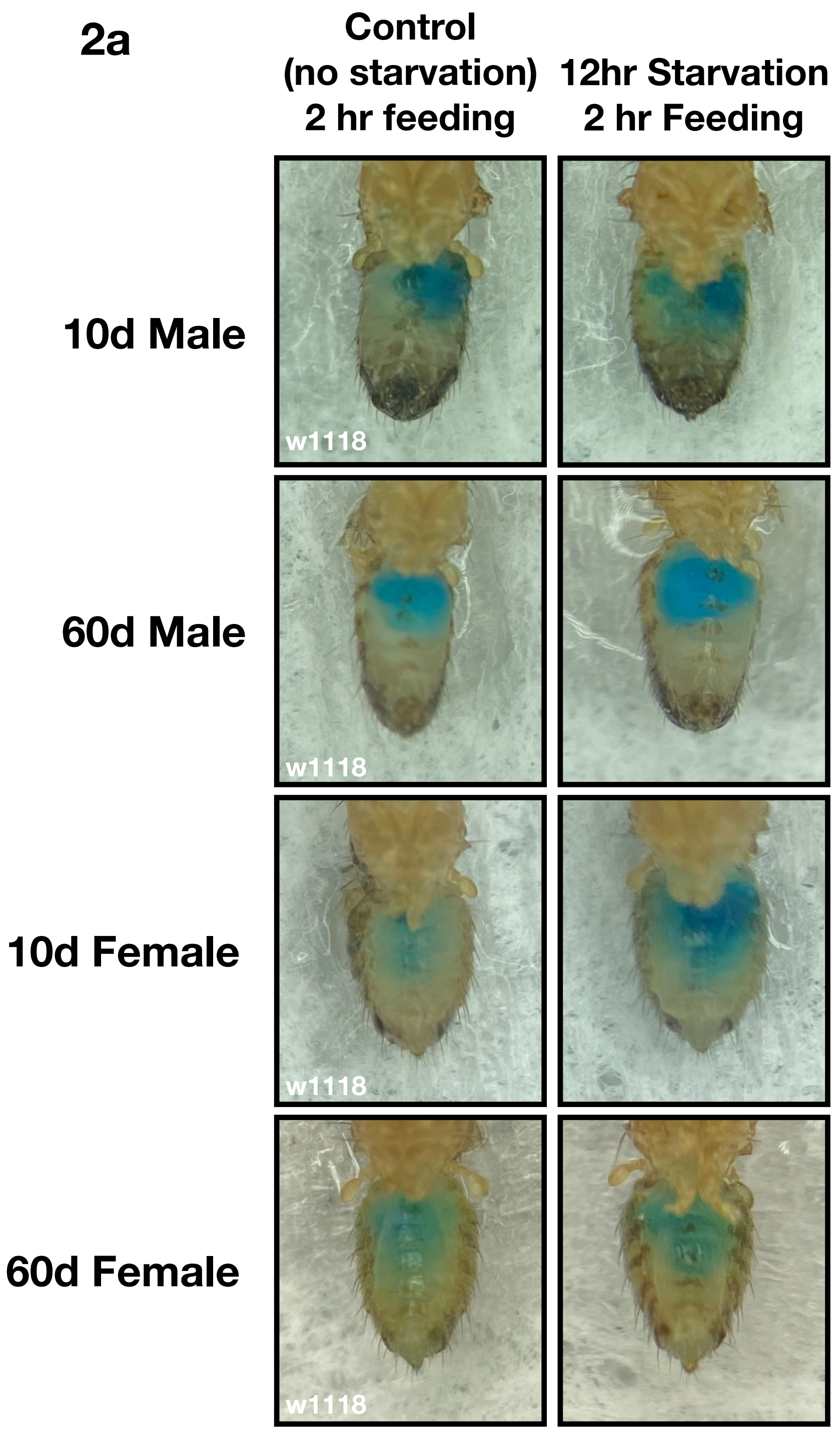

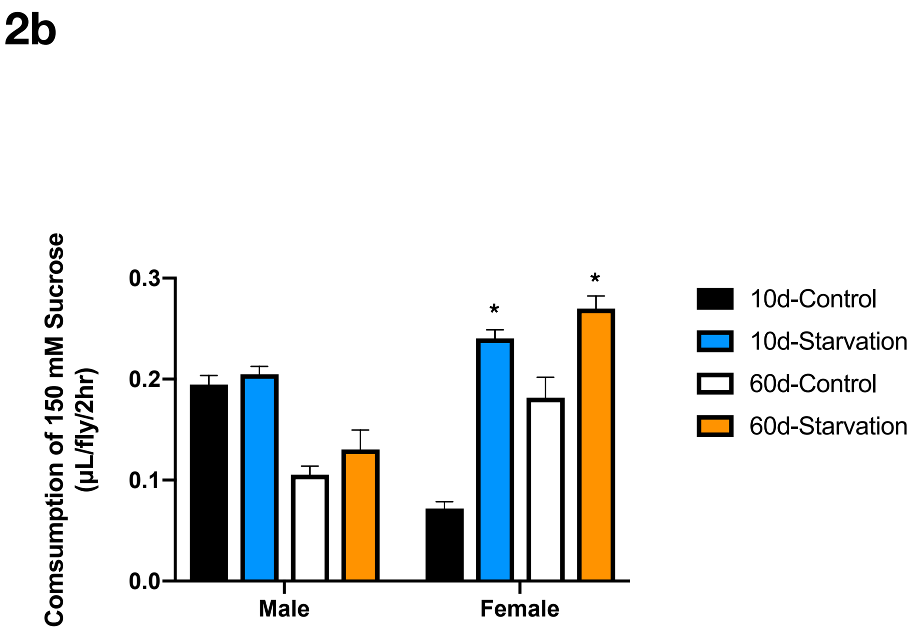

Supplementary Figure 3.

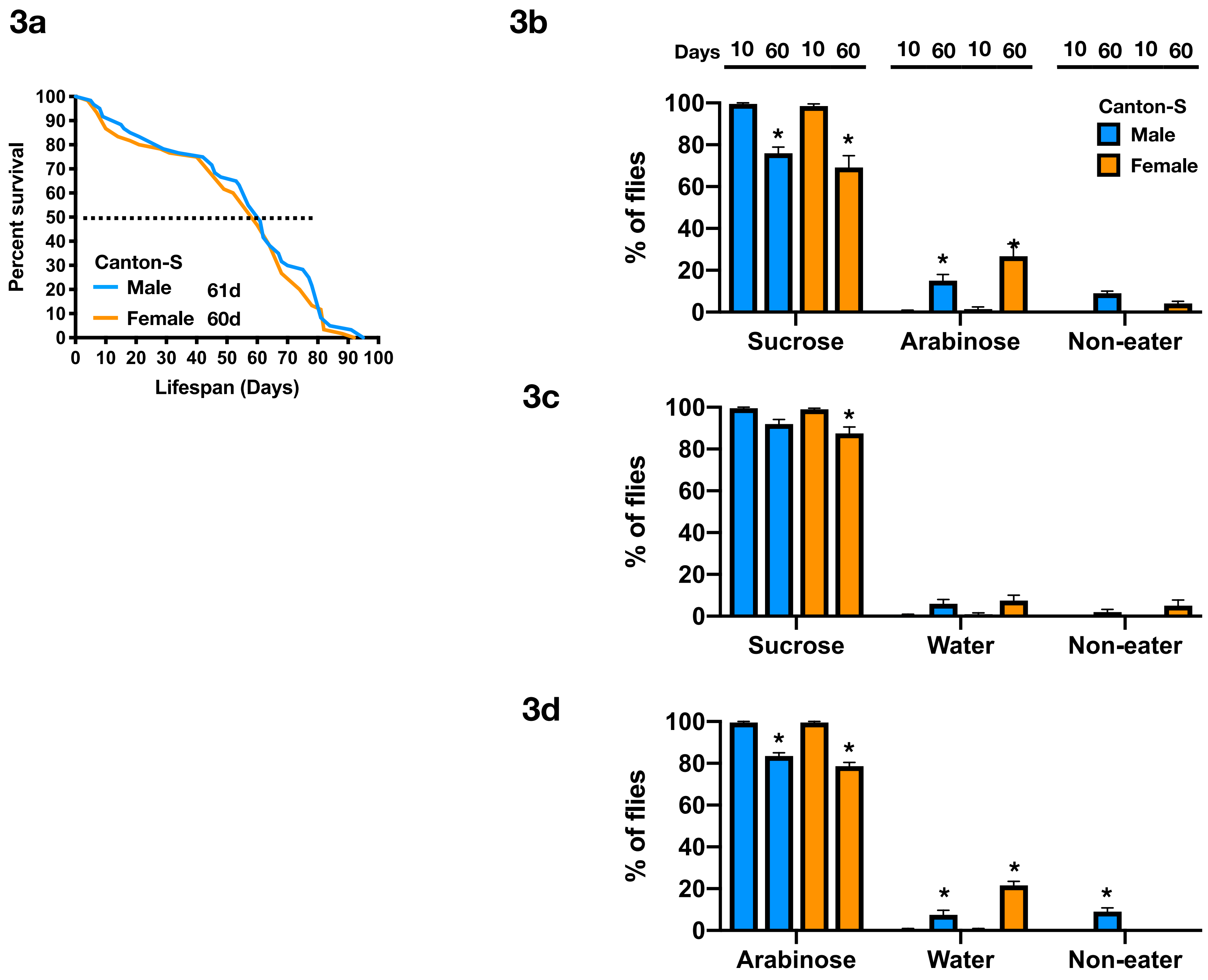

Supplementary Figure 4.

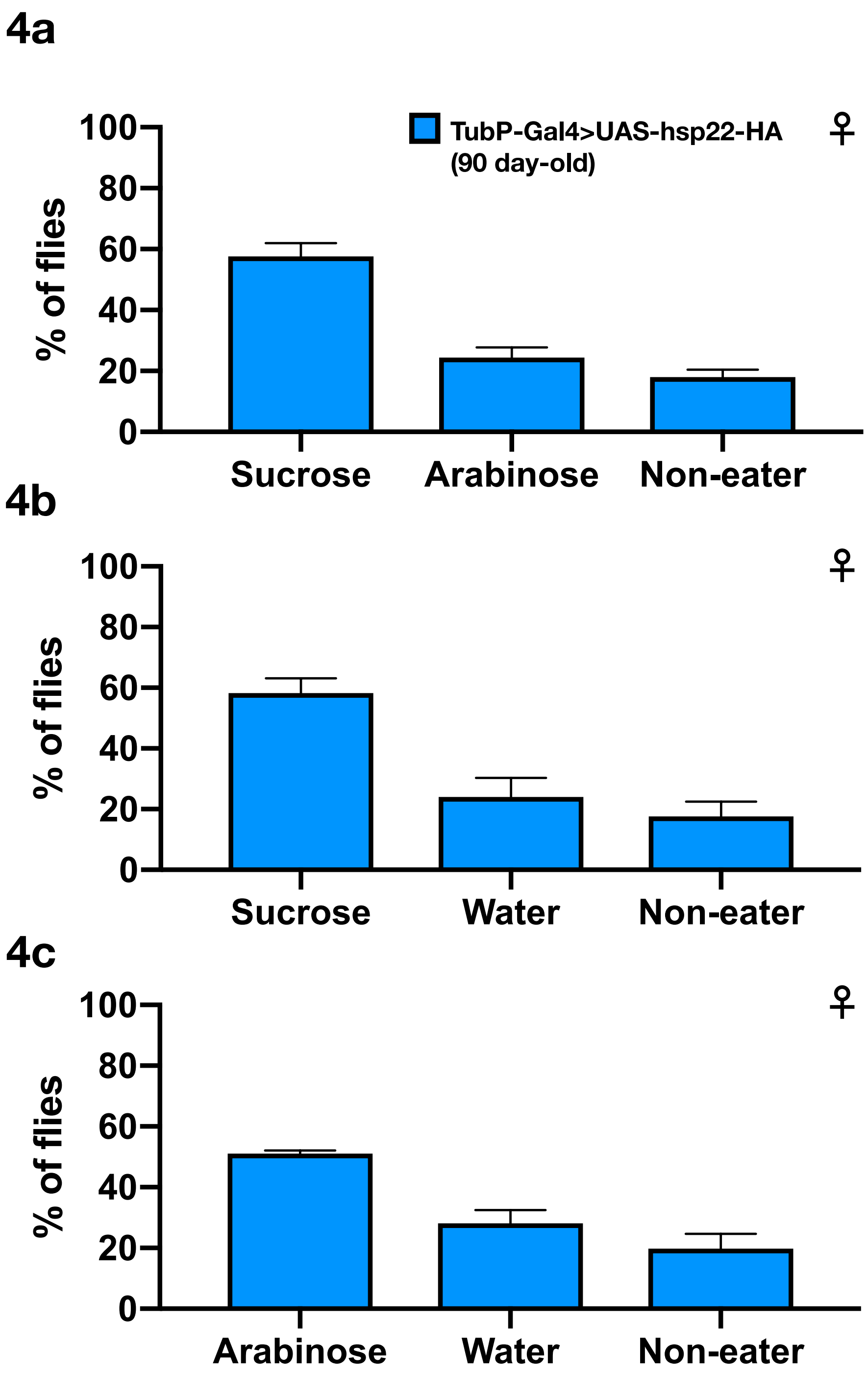

Supplementary Figure 4.

4d

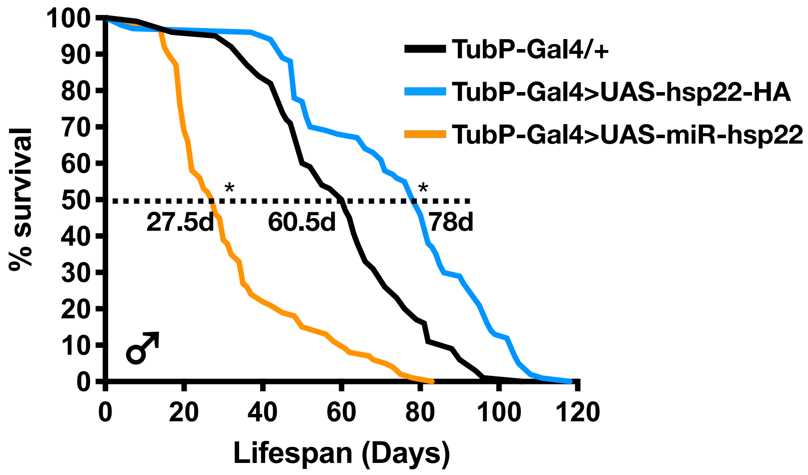

4e

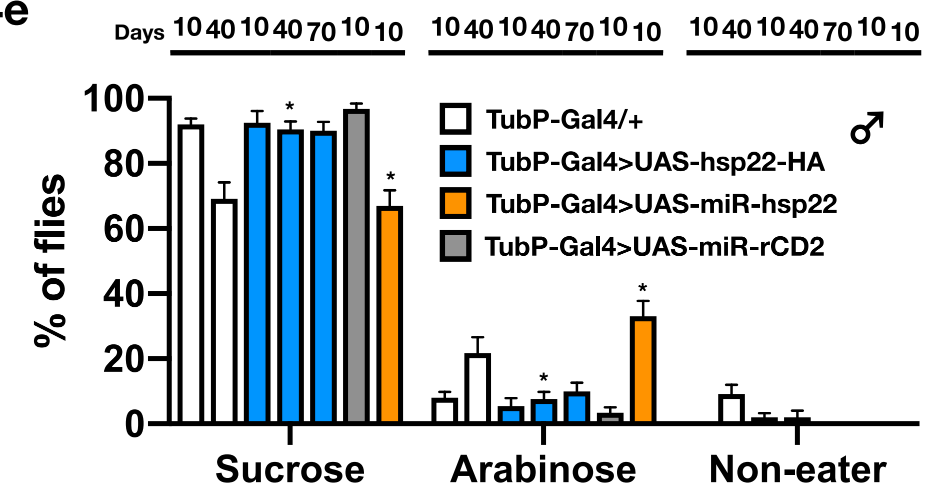

4f

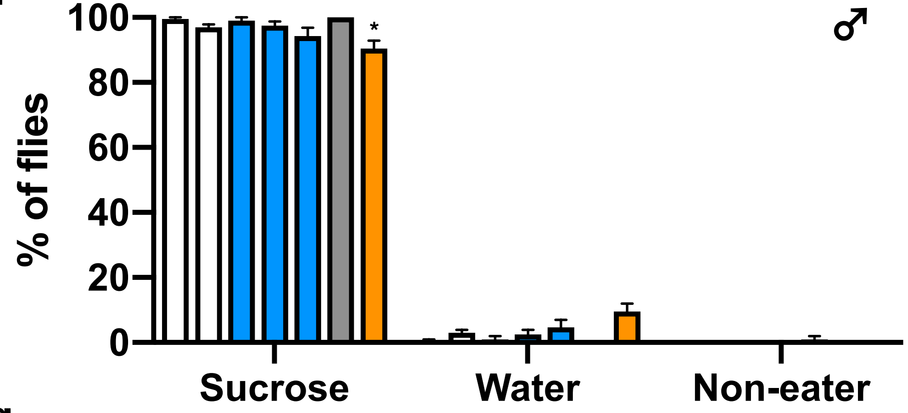

4g

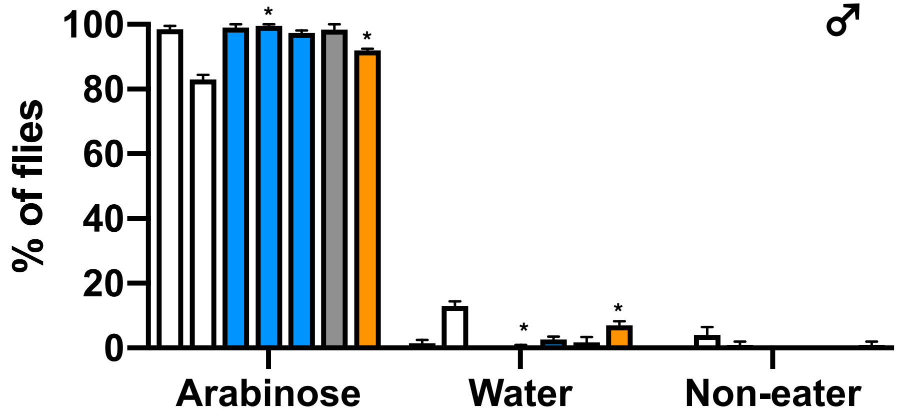

Supplementary Figure 5.

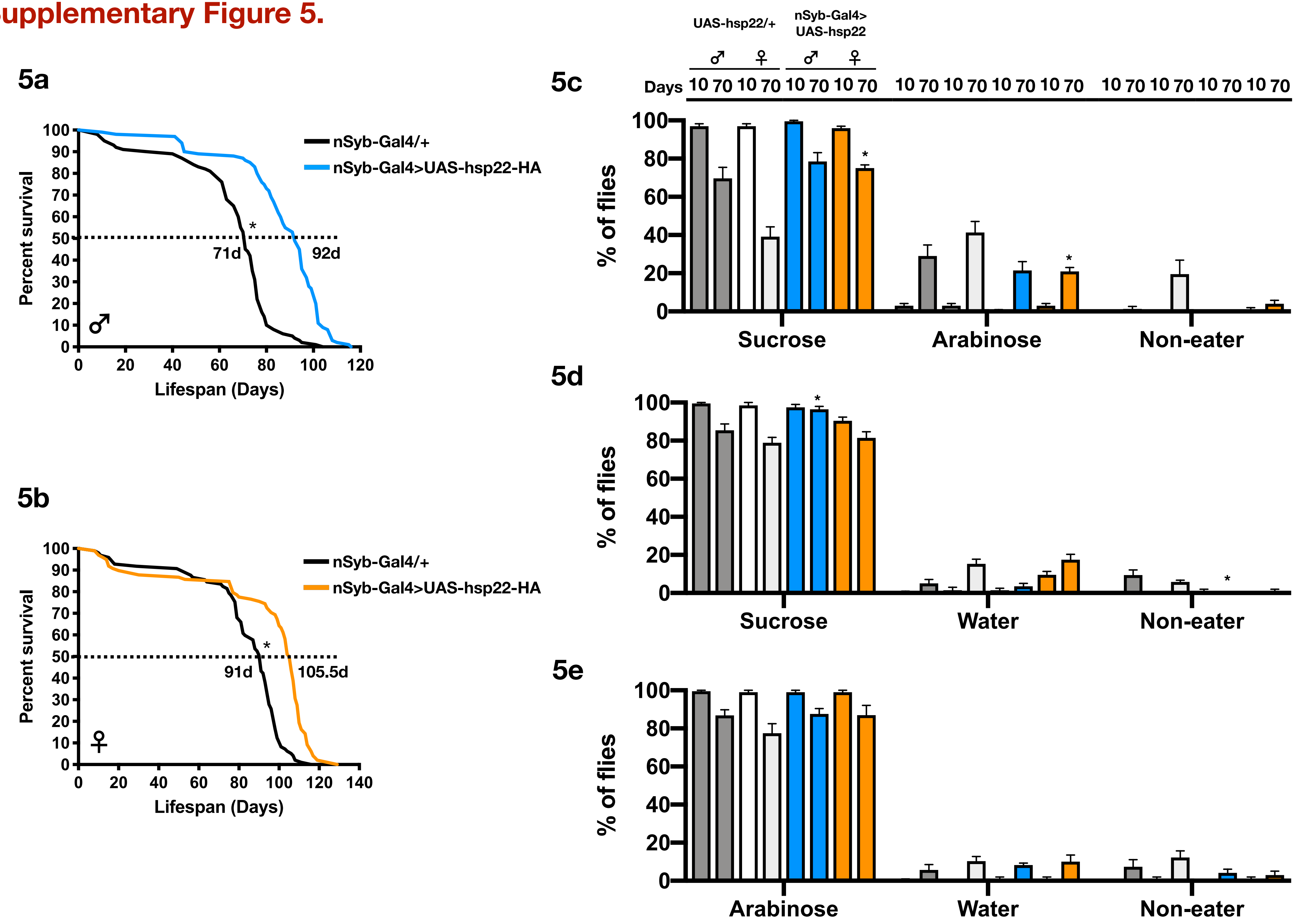

Supplementary Figure 6.

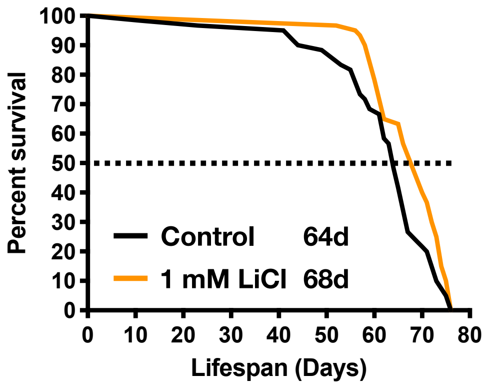

Supplementary Figure 7.

7a

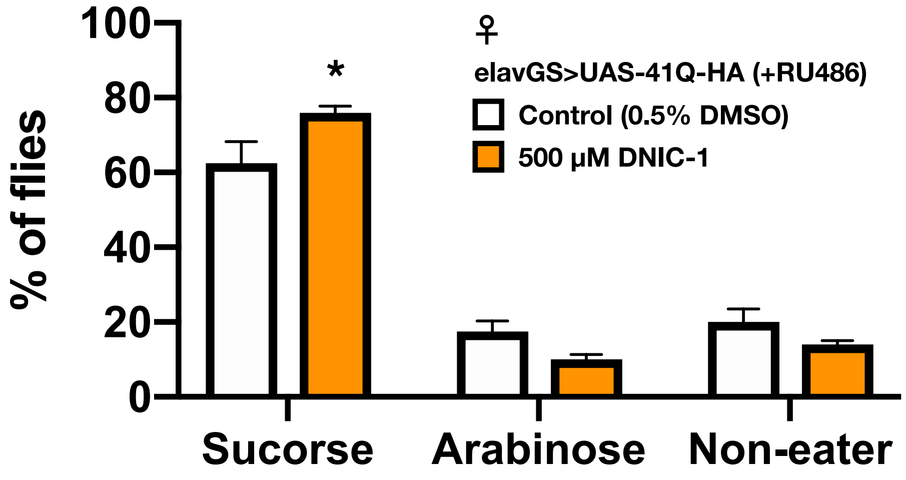

7b

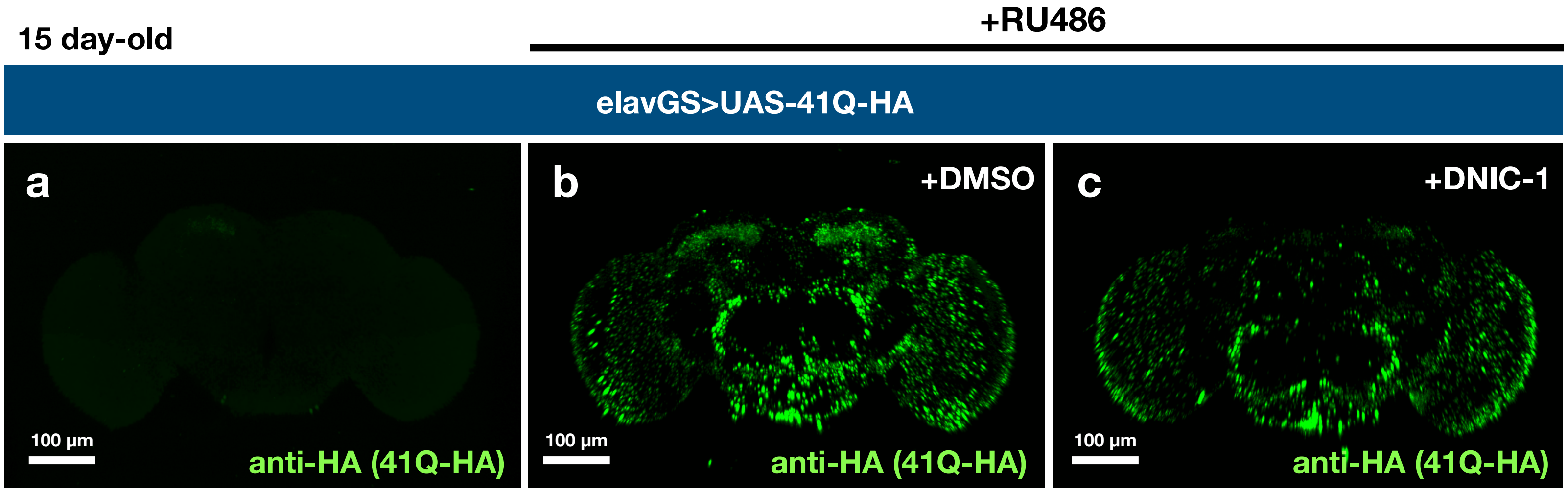

Supplementary Figure 8.

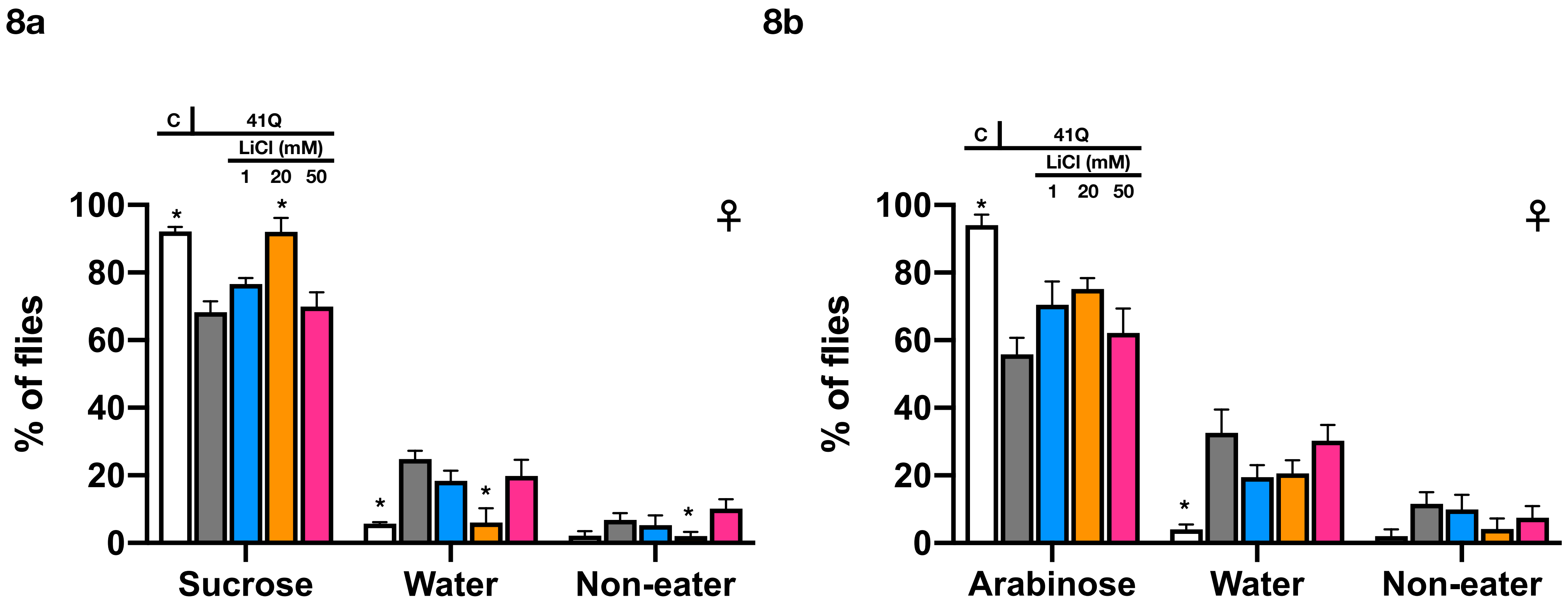

Supplementary Figure 9.

9a

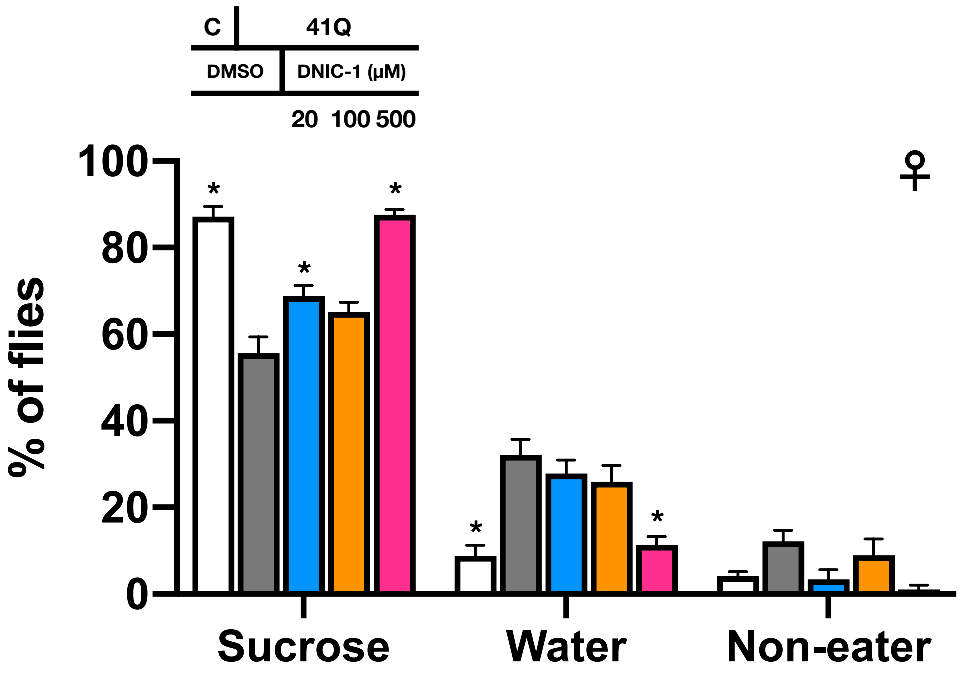

9b

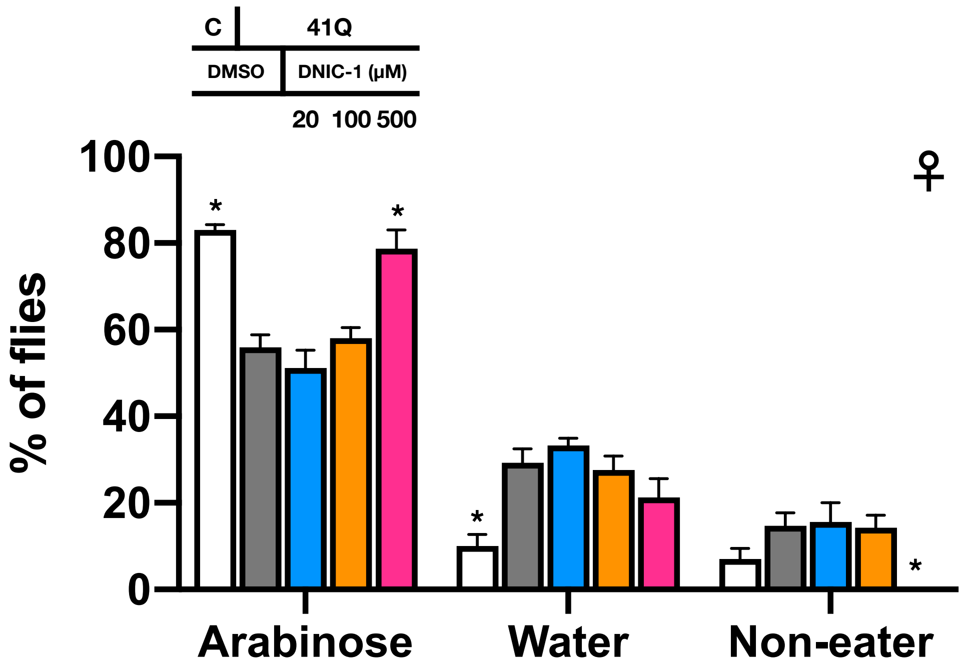

9c

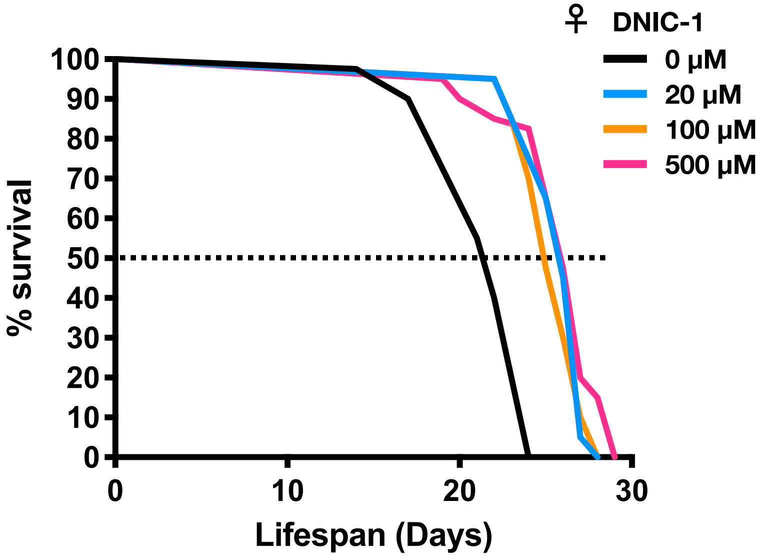

DNIC-1	Median Lifespan (Days)	Maximum Lifespan (Days)	n=
0 μM	22	24	40
20 μM	26	28	20
100 μM	25	28	40
500 μM	26	29	40

Supplementary Figure 9.

9d

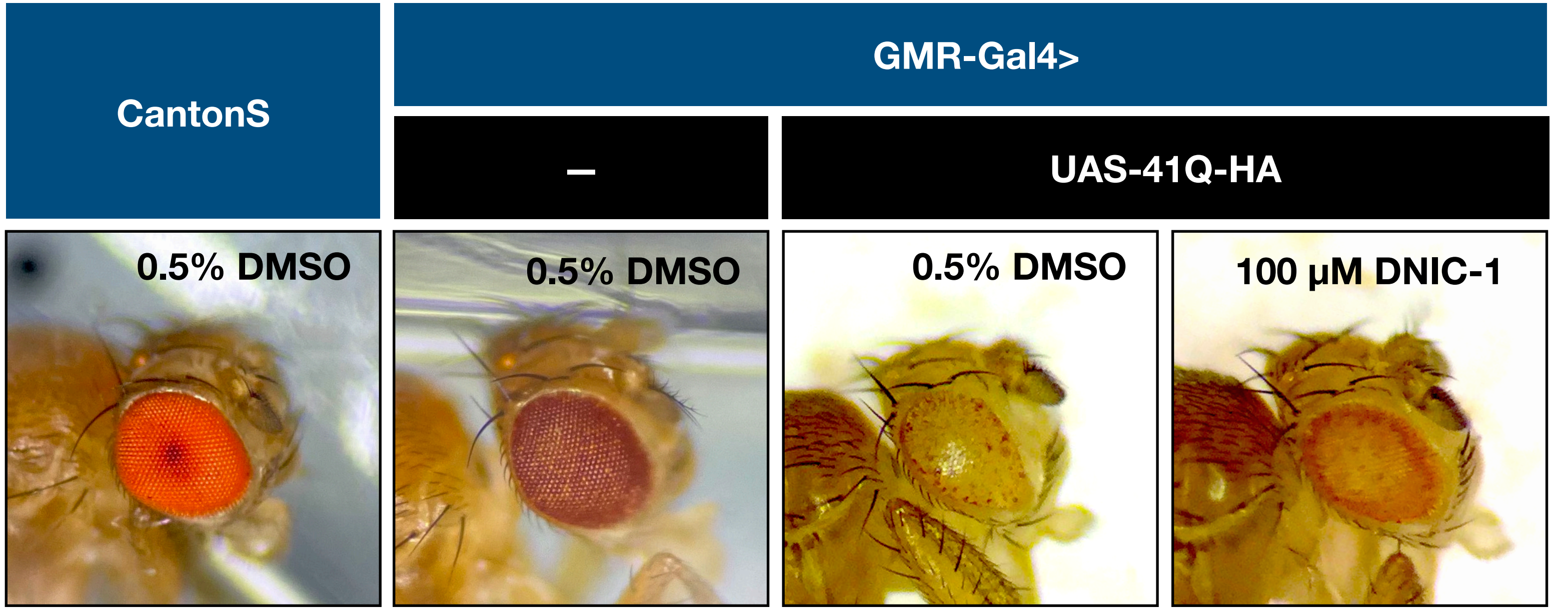

Supplementary Figure 9.

9e

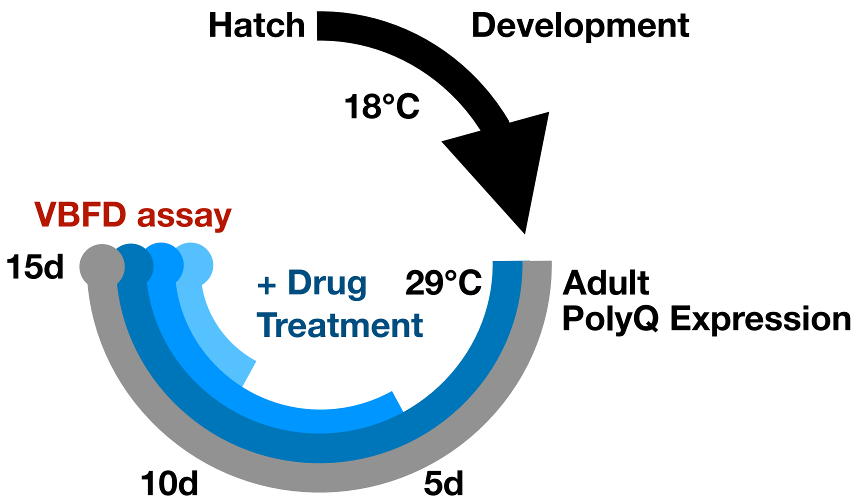

9f

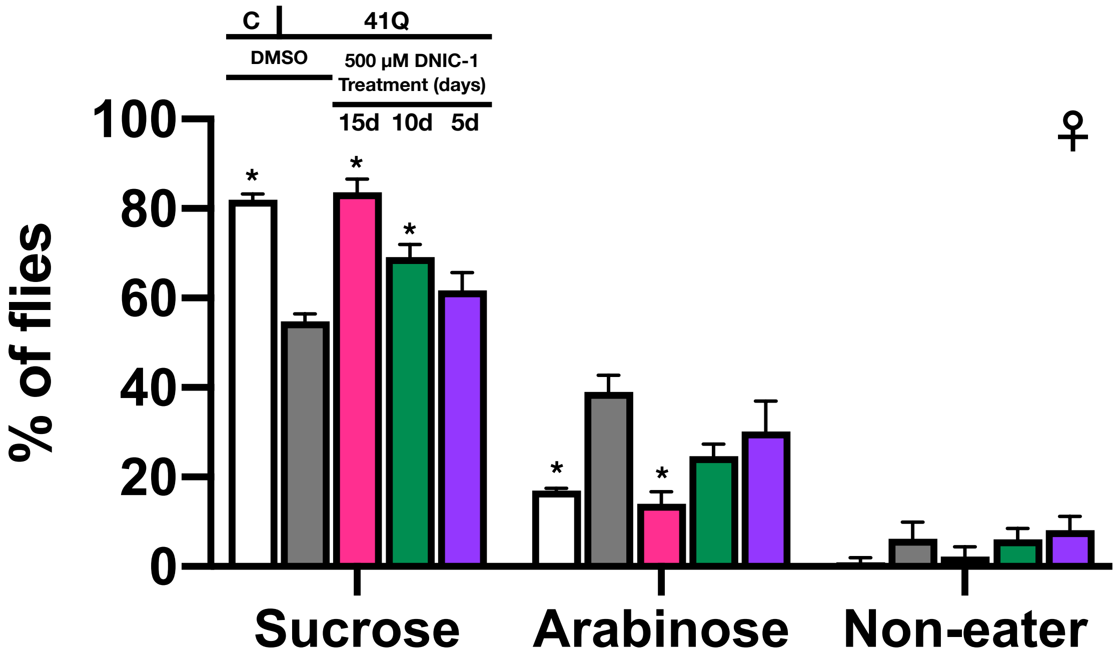

Supplementary Figure 9.

9g

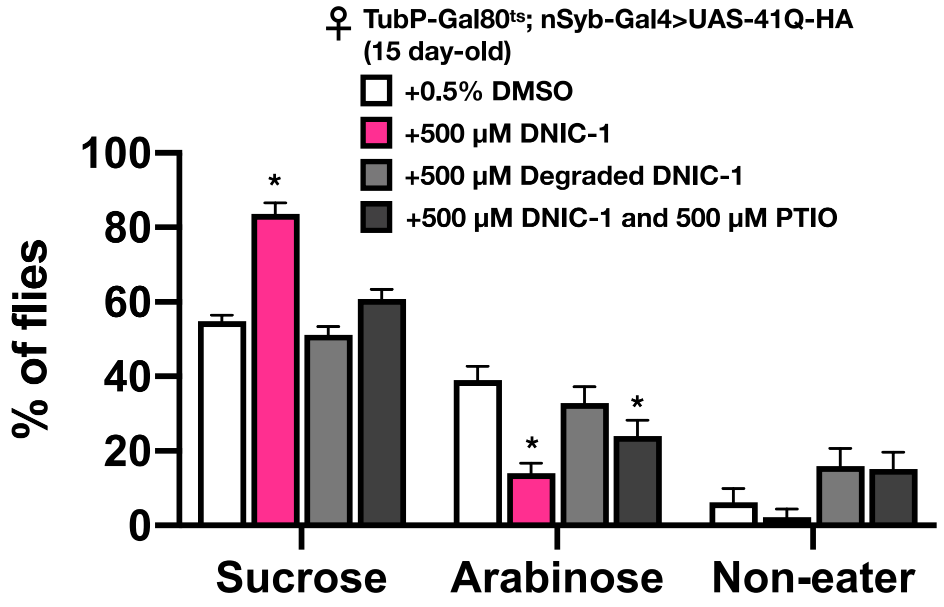

9h

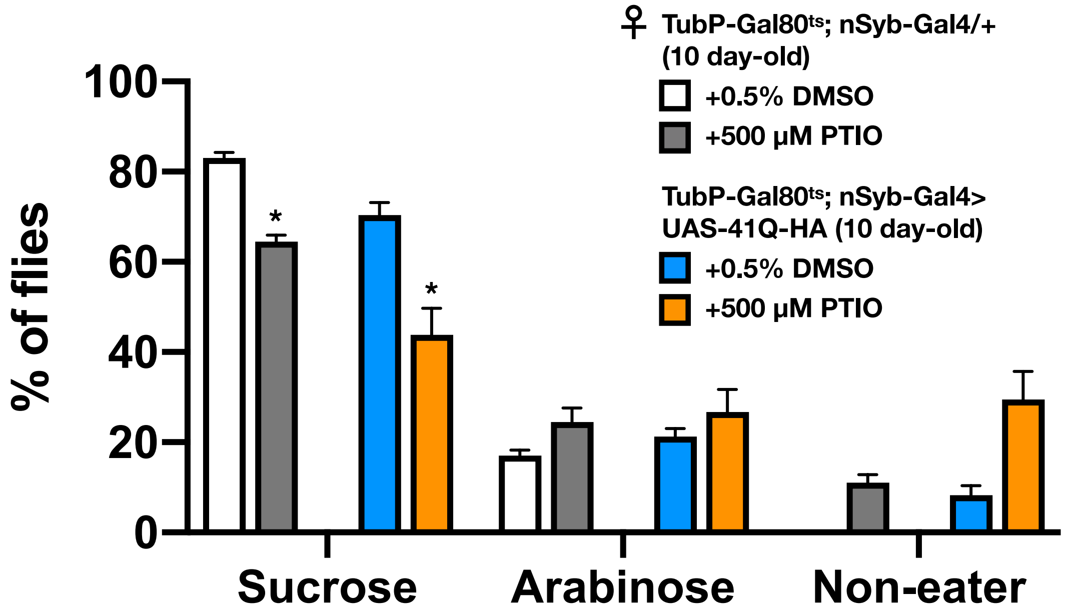

9i

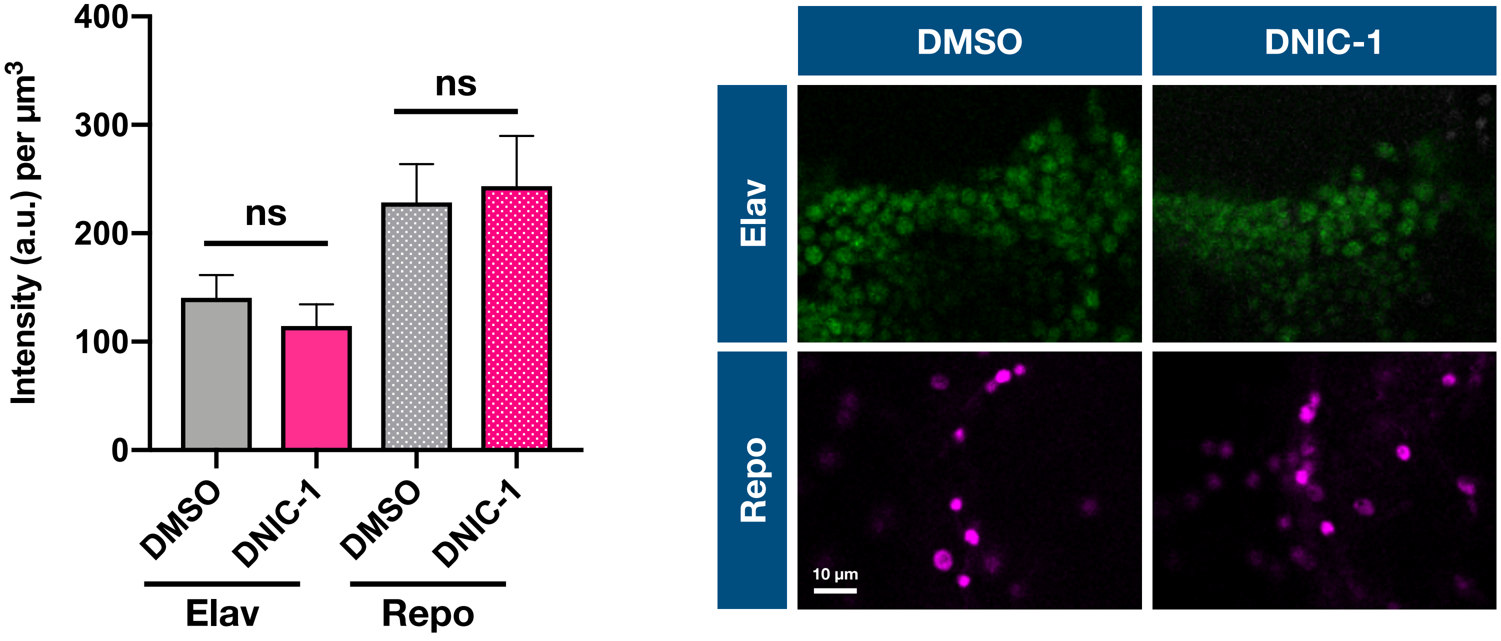

Supplementary Figure 9.

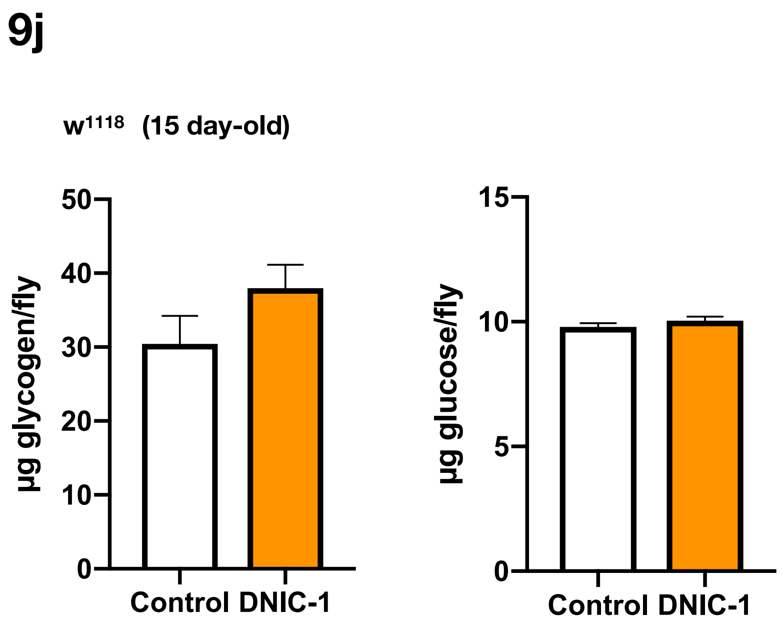

Supplementary Figure 10.

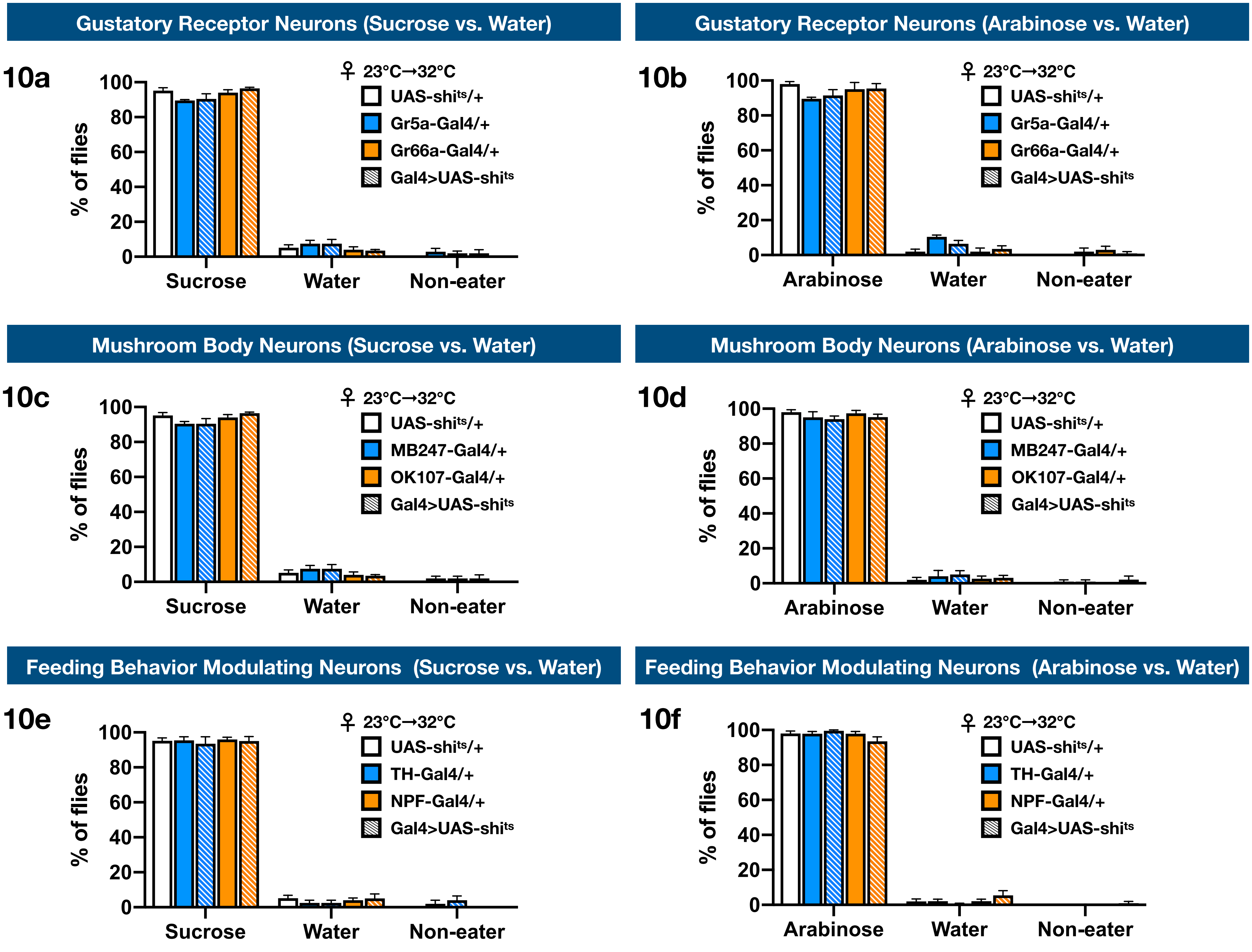

Supplementary Figure 10.

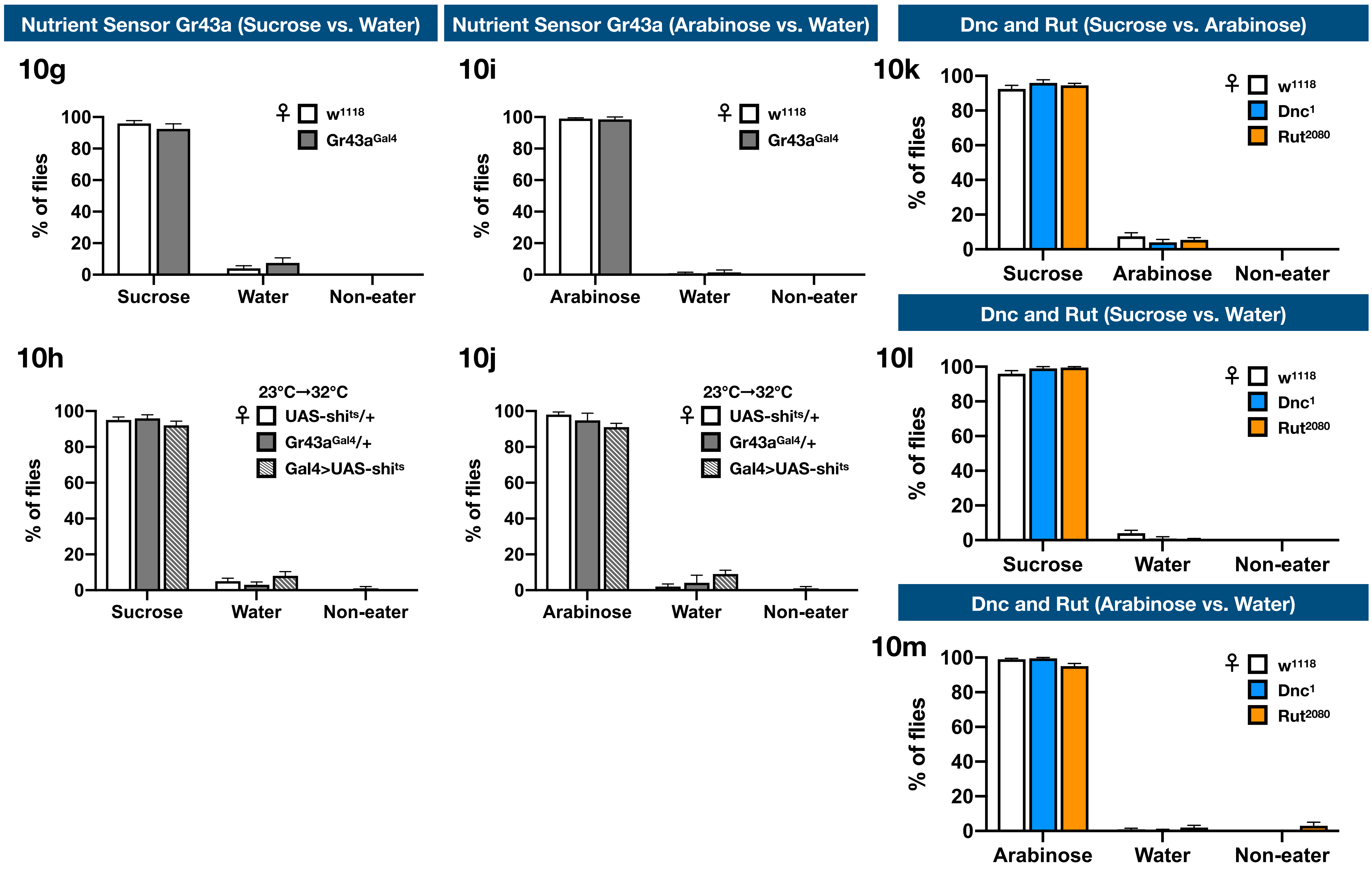

Supplementary Figure 11.

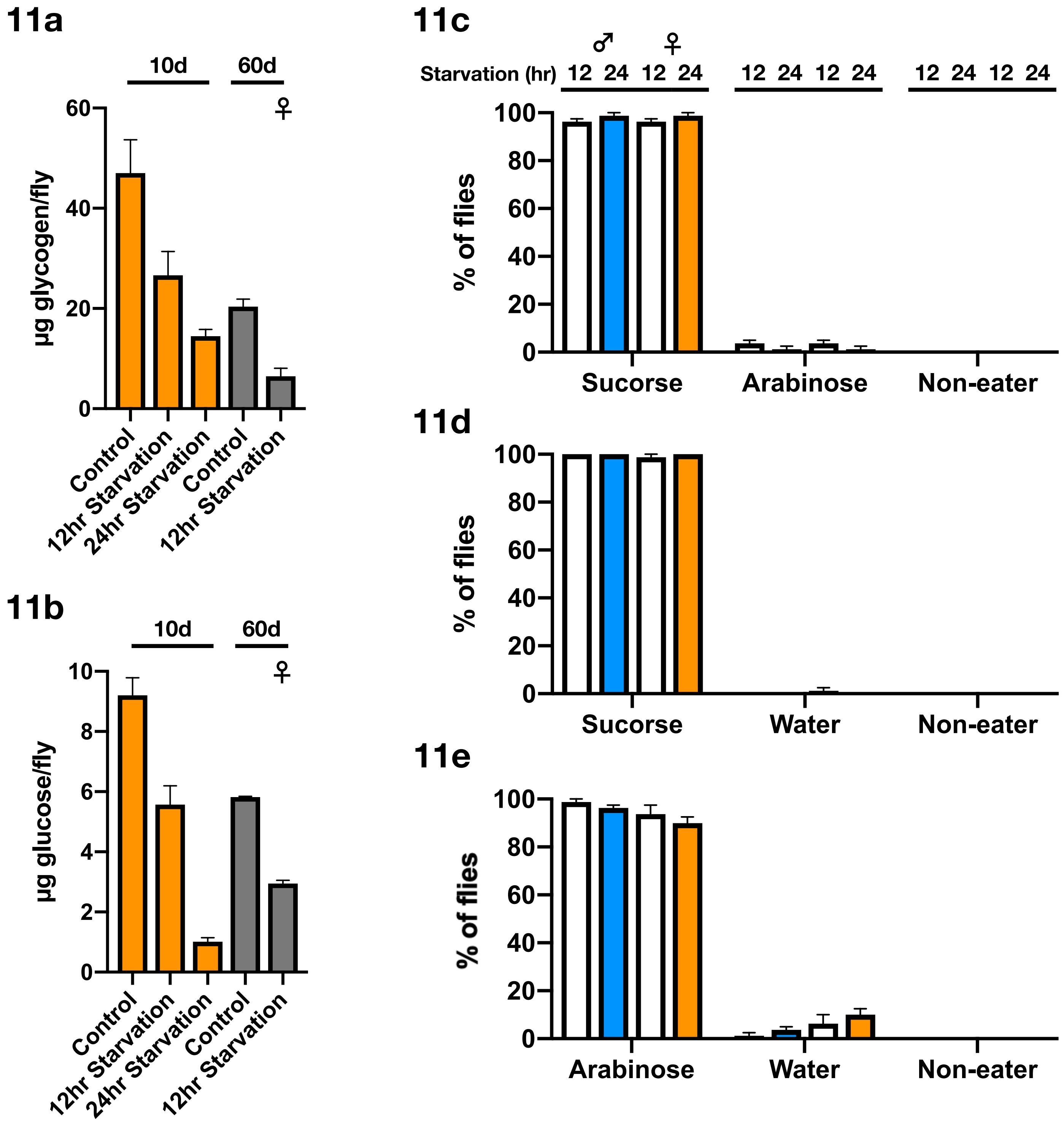

Supplementary Figure 12.

12a

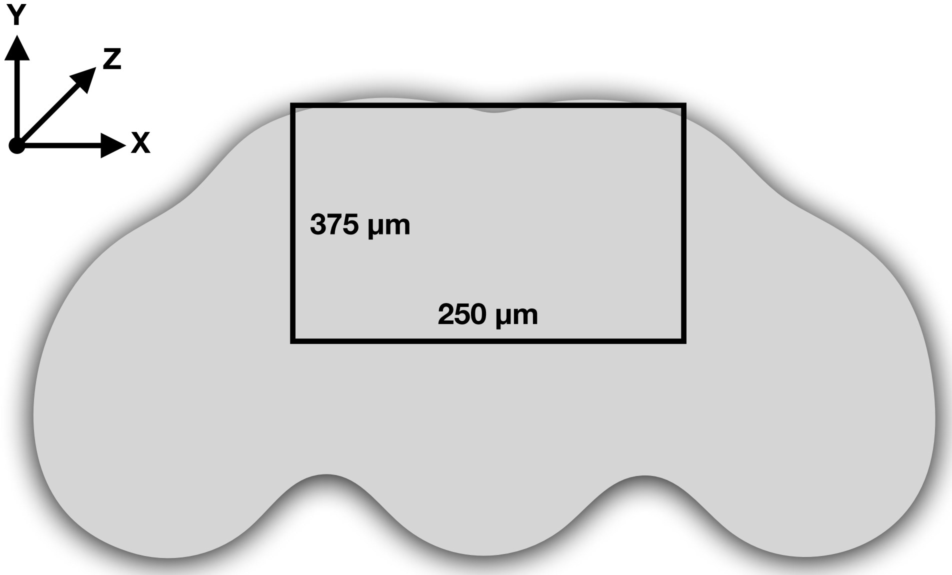

Z: brain thickness

12b

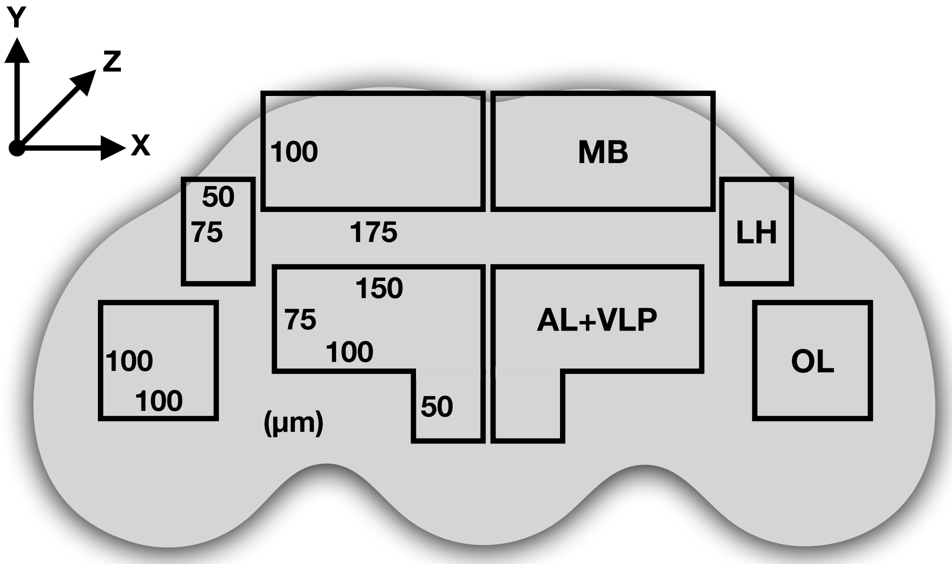

Z: brain thickness

Z of MB: 1/2 brain thickness

Table S1. List of primer sequences used for generating UAS-hsp22-HA and UAS-miR-hsp22 lines

Primers	Sequence 5'-3'
Hsp22-HA forward	ggggaattcatgcgttccttaccgatgttttggcgcatg
Hsp22-HA reverse	gggctcgagctaagcgtaatctggaacatcgtatgggtactgactggcggctttgtcatttggctc
Hsp22-mir-1-1 forward	ggcagcttacttaaacttaatcacagcctttaatgtcaagaagtatttcaaagacaactaagttaatataccatatc
Hsp22-mir-1-2 reverse	aataatgatgttaggcactttaggtaccaagaagtatttcaaagacaactagatatggtatattaacttagttg
Hsp22-mir-2-1 forward	ggcagcttacttaaacttaatcacagcctttaatgtctagtgaactcctatgtttagataagttaatataccatatc
Hsp22-mir-2-2 reverse	aataatgatgttaggcactttaggtacctagtgaactcctatgtttagatagatatggtatattaacttatcta
Mir6.1_5’EcoRI/BglII forward	ggcgaattccgccagatcttttaaagtccacaactcatcaaggaaaatgaaagtcaaagttggcagcttacttaaactta
Mir6.1_3’BamHI/NotI reverse	ggccgcggccgcacggatccaaaacggcatggttattcgtgtgccaaaaaaaaaaaaaattaaataatgatgttaggcac
